# Impact of the Gas Atmosphere at the Triple Boundary Phase on the Measured Oxygen Evolution Reaction Activity of Ni_2_B/Ni_3_B Electrocatalysts

**DOI:** 10.1002/smtd.202501880

**Published:** 2025-12-04

**Authors:** Lithin Madayan‐Banatheth, Alejandro E. Perez‐Mendoza, Ulrich Burkhardt, Iryna Antonyshyn, Corina Andronescu

**Affiliations:** ^1^ Chemical Technology III University of Duisburg‐Essen Carl‐Benz‐Straße 199 47057 Duisburg Germany; ^2^ Max‐Planck‐Institut für Chemische Physik fester Stoffe Nöthnitzer Straße 40 01187 Dresden Germany; ^3^ Fritz‐Haber‐Institut der Max‐Planck‐Gesellschaft Faradayweg 4–6 14195 Berlin Germany; ^4^ CENIDE, Center for Nanointegration University of Duisburg‐Essen Carl‐Benz‐Straße 199 47057 Duisburg Germany

**Keywords:** crystal structure, nano electrochemistry, nickel borides, oxygen evolution reaction, scanning electrochemical cell microscopy, structure ‐ activity correlations

## Abstract

The evaluation of the oxygen evolution reaction (OER) electrocatalytic activity of established electrocatalysts is key for the rational design of new electrocatalysts. In this study, scanning electrochemical cell microscopy (SECCM) is employed to assess, under identical conditions, the OER electrocatalytic activity of Ni_2_B and Ni_3_B, simultaneously present in a Ni_70_B_30_ ingot. An important finding is that the gas environment surrounding the nanodroplet formed at the tip of the SECCM probe and the Ni_70_B_30_ ingot impacts the measured current density. The presence of a gas flowing (air, Ar, CO_2_, and O_2_) outside the electrolyte droplet increases the measured OER current recorded on both phases, compared to a situation without gas convection, revealing one of the key parameters that can be used to enable higher OER current densities to be recorded on the same catalyst. Notably, the presence of CO_2_, even in small concentrations (2% O_2_ in Ar) in the surrounding atmosphere, leads to a significant apparent decrease of the OER activity. The study reveals that Ni_3_B shows an almost 20% enhanced OER electrocatalytic activity compared to Ni_2_B, which contradicts previous findings and highlights the importance of precisely controlled experiments enabled by SECCM when establishing catalytic trends.

## Graphical abstract

This study reveals the impact of gas convection and its composition on the oxygen evolution reaction (OER) electrocatalytic activity of Ni‐B catalysts using scanning electrochemical cell microscopy (SECCM). Applying a controlled protective Ar atmosphere, and using a well‐defined Ni‐B specimen, containing Ni_3_B and Ni_2_B phases, it is revealed that Ni_3_B has an increased OER activity compared to Ni_2_B.

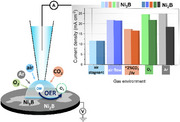

## 1 Introduction

The conversion or evolution of a gaseous compound is a part of almost all electrochemical reactions that are important for energy conversion and storage.^[^ ^1^, ^2^, ^3^, ^4^ ^]^ Hydrogen or oxygen generation via electrocatalytic water splitting,^[^ ^1^ ^]^ or carbon dioxide electroreduction to produce ethylene or ethanol,^[^ ^3^ ^]^ are just a few examples. Besides the gaseous species consumed or formed, these reactions also require the transfer of protons, often provided by the electrolyte. Thus, the electrochemical reactions often involve the presence of a three‐phase boundary (TPB) governed by dynamic processes in which mass transport effects impact the system performance.^[^ ^2^ ^]^ Previous studies revealed that by optimizing the TPB, a significant boost in the overall reaction efficiency for hydrogen oxidation and oxygen reduction reactions was observed.^[^ ^5^, ^6^ ^]^ This highlights the importance of understanding the microenvironments formed during gas‐involving reactions and their impact on the overall reaction rate to design more efficient electrocatalysts.

When macroelectrodes are tested for gas‐evolving reactions, the accumulation of the gas product at the electroactive surface triggers the formation of gas bubbles, which may hinder the diffusion of reactants and products and reduce the electrical conductivity.^[^ ^7^, ^8^, ^9^, ^10^ ^]^ Even for nano‐impact electrochemistry measurements allowing the enhanced mass transport due to the increased contribution of the radial diffusion, it was shown that the measured catalytic activity is influenced by the removal of formed O_2_ during the reaction.^[^ ^11^ ^]^

Scanning electrochemical cell microscopy (SECCM), in which an electrochemical cell is formed by contacting a nm‐sized hanging electrolyte droplet located at the tip of a nanopipette on a catalyst surface, was successfully used to derive structure‐activity correlations for several catalysts^[^ ^12^, ^13^, ^14^ ^]^ used in gas‐evolving reactions with decreased impact of the mass transport limitation on the recorded current.^[^ ^12^, ^15^, ^16^ ^]^ This was possible due to the formation of a TPB in SECCM, which facilitates the fast removal of gaseous products from the surface, enabled by the short distance between the catalyst surface to the gas/meniscus interface (maximum diffusion length given by the radius of the nanodroplet).^[^ ^16^, ^17^ ^]^ Recently, using finite element simulation (FES), it was shown that the interfacial gas transfer kinetics impact the measured electrode kinetics for the hydrogen evolution reaction (HER), used as a model for gas‐evolving reactions.^[^ ^18^ ^]^ However, evaluating the oxygen evolution reaction (OER) activity of catalysts using SECCM in alkaline electrolytes meets additional challenges, especially in controlling the droplet size and, thus, in normalizing the measured currents to the probed area.^[^ ^19^, ^20^ ^]^ Therefore, the number of papers in which SECCM is used to evaluate the OER electrocatalytic activity of catalysts is still limited compared with those dedicated to the local HER electrocatalytic activity of different catalysts.

Ni‐based catalysts are among the most investigated materials for alkaline water electrolysis.^[^ ^21^, ^22^, ^23^ ^]^ Among them, Ni borides have great potential, showing superior electrocatalytic activity compared to elemental Ni, frequently explained by a reverse electron transfer,^[^ ^24^, ^25^ ^]^ a hypothesis that is still under debate. The impact of B content on OER activities of Ni‐B electrocatalysts was addressed in several publications using particle‐based catalyst films of Ni‐B having not only different Ni:B contents but also different structures and/or morphologies.^[^ ^25^, ^26^ ^]^ The improved electrocatalytic activity of Ni‐B catalysts was explained by an enhanced surface oxidation with the formation of borates and nickel oxide layers on the surface of Ni_x_B when exposed to air.^[^ ^25^, ^27^, ^28^, ^29^ ^]^ Furthermore, recent studies indicate that Fe impurities present in the electrolyte can have a noticeable impact on the electrocatalytic activity of Ni‐B electrocatalysts.^[^ ^30^ ^]^ All these results indicate that to achieve a deeper fundamental understanding of these materials, there is a need for model well‐defined Ni‐B electrocatalysts, as well as for controlled electrochemical measurements that allow the probing of the electrocatalytic activity, in the absence of film effects or mass transport limitations in a controlled environment. Herein, we aim to evaluate the OER electrocatalytic activity of Ni_2_B and Ni_3_B phases coexisting in the Ni_70_B_30_ ingot using identical conditions enabled by SECCM. To achieve this, we focused on understanding how the gas atmosphere involved in the TPB formed during SECCM measurements impacts the measured OER electrocatalytic activity of Ni‐B catalysts in the alkaline electrolyte.

## 2 Results and Discussion

**Figure 1:**
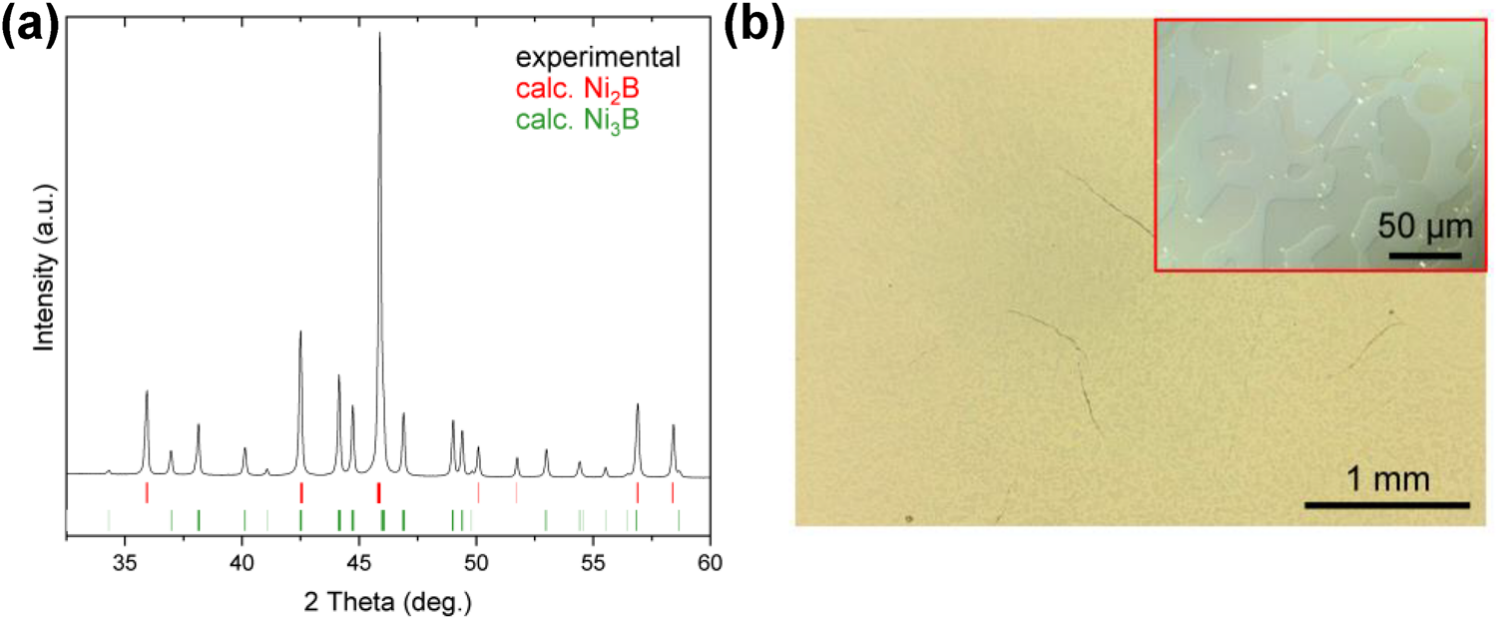
a) Powder X‐ray diffraction pattern of two‐phase sample Ni_70_B_30_ (black curve) superimposed with the calculated patterns of Ni_2_B (red ticks) and Ni_3_B (green ticks). b) Light microscopy image of Ni_70_B_30_ in bright field and selected magnified area with differential interference contrast (inset), revealing homogeneous distribution of Ni_2_B and Ni_3_B phases in the specimen.

According to the phase diagram of the binary Ni‐B system,^[^ ^31^ ^]^ a phase equilibrium exists between the two phases, Ni_2_B and Ni_3_B. Therefore, to obtain the sample with roughly equal amounts of both phases, the sample was prepared at the composition Ni_70_B_30_ (for synthesis details – see Section S1.1, Supporting Information). Powder X‐ray diffraction data of the synthesized sample clearly reveal the presence of two crystalline phases: Ni_2_B (CuAl_2_ type of structure, space group *I*4/*mcm*)^[^ ^32^ ^]^ and Ni_3_B (Fe_3_C structure type, space group *Pnma*)^[^ ^33^ ^]^ (**Figure** **1**a). The refined lattice parameters (*a*  =  4.9908(2) Å, *c*  =  4.2491(2) Å for Ni_2_B and *a*  =  5.2216(2) Å, *b*  =  6.6162(2) Å, *c*  =  4.3907(2) Å for Ni_3_B) are very close to those published in the literature (Ni_2_B: *a*  =  4.991(3) Å, *c*  =  4.247(3) Å,^[^ ^32^ ^]^ Ni_3_B: *a*  =  5.2219(2) Å, *a*  =  6.6171(2) Å, *c*  =  4.3918(1) Å^[^ ^33^ ^]^). This confirms the successful synthesis and proximity of the elemental compositions of both borides to the intended ones. Likewise, the elemental ratios of nickel to boron quantified using wavelength‐dispersive X‐ray spectroscopy in the Ni_70_B_30_ sample are Ni:B  =  75.2(2) : 24.8 (at. %) for Ni_3_B and Ni:B  =  66.5(6) : 33.5 (at. %) for Ni_2_B phases. The overview light microscopy image of Ni‐B sample as well as the differential interference contrast picture, clearly reveal the homogeneous distribution of both phases throughout the sample (Figure 1b).

Localized electrochemical measurements were performed using SECCM (Figure S1, Supporting Information) in the hopping mode to evaluate the local OER electrocatalytic activity of two Ni‐B phases. Initially, the OER activity was measured in the surrounding air (without any gas flow), using a nanopipette having a ≈590 nm tip (Figure S2a, Supporting Information) filled with 0.1 M KOH. Linear sweep voltammograms (LSVs) were recorded on each landing point in the potential window of 0.27 to 1.90 V versus RHE at a scan rate of 1 V s^−1^. To enable further comparison, the currents were normalized to the area of the footprint left by the nanodroplet on the catalyst surface (Figure S4a, Supporting Information), calculated from SEM images (Figure S3 and Section S1.4, Supporting Information). The recorded LSVs present an initial oxidation process starting at ≈0.8 V versus RHE, which indicates a possible reconstruction or transformation of Ni‐B surfaces, followed by the oxidation of Ni^2+^ to NiOOH observed at ≈1.5 V versus RHE.^[^ ^34^ ^]^ A continuous increase in the current at higher anodic potentials is observed, with a change in the slope after 1.7 versus RHE, attributed to OER (Figure S4c, Supporting Information). The current density maps derived at 1.8 V versus RHE (Figure S4b, Supporting Information) and the corresponding histograms (Figure S4d, Supporting Information) show no clear differences in the OER electrocatalytic activity recorded across the Ni–B surface. Comparison with the SEM image shows that the Ni_3_B regions (appearing with lighter contrast) exhibit similar current density intensity and distribution to the Ni_2_B regions (darker contrast). These results contradict previous reports from the literature that indicate Ni_2_B as being more active in OER compared to Ni_3_B.^[^ ^25^, ^26^ ^]^

A further analysis of the current densities recorded as a function of the footprints left by the SECCM tip on each measured area (MA) of the Ni_2_B and Ni_3_B phases was conducted (Figure S4e, Supporting Information). Interestingly, a linear correlation can be established between the current densities and the footprint. An increase in current density from ≈10.1 up to ≈13.6 mA cm^−2^ occurs at 1.8 V versus RHE when the wetted area decreases from 1.52 to ≈1.05 µm^2^. This linear trend may be attributed to the enhanced mass transport of formed O_2_ from the catalyst surface, due to an increased ratio between the liquid/gas interface and the liquid volume, and indicates that the size of the formed electrochemical cell impacts the measured current. This observation is consistent with the behavior expected for nanoelectrodes: as the electrode size decreases, the diffusion field becomes increasingly radial (hemispherical), resulting in shorter diffusion paths and higher steady‐state fluxes.^[^ ^35^ ^]^ This trend aligns with a recent study reporting enhanced mass transfer rates during the hydrogen evolution reaction (HER) with decreasing footprint size, particularly pronounced for footprints below 300 nm.^[^ ^18^ ^]^ This geometric dependence has also been experimentally verified by Schuhmann et al.,^[^ ^16^ ^]^ who systematically compared SECCM probes with diameters of 3.78, 1.29 µm, 406, and 155 nm, and demonstrated that the maximum current density varies inversely with probe size as a result of the increasing dominance of radial diffusion at smaller footprints. On the other hand, gas transport can happen both ways, from and to the hanging nanodroplet over the liquid/gas interface, and thus, we cannot neglect that the O_2_ present in air may impact this transport. Already at macroscale experiments conducted using a rotating disk electrode, it was shown that the presence of O_2_ in the electrolyte leads to a decrease in the OER current on the Ni(OH)_2_ catalyst. This decrease was more pronounced in the absence of rotation^[^ ^36^ ^]^ and was explained by the accumulation of O_2_ close to the surface, which may promote the formation of tiny bubbles and changes in the surface. Besides O_2_, other gases such as CO_2_ are present in low concentrations in the air (421 ppm).^[^ ^37^ ^]^ In particular, in an alkaline‐based electrolyte, CO_2_ can be captured from air and can consume the OH^−^ in the electrolyte to form HCO_3_^−^/CO_3_^2−^, which leads to a decrease in the pH of the electrolyte.^[^ ^38^, ^39^, ^40^, ^41^ ^]^ For oxygen reduction reaction (ORR), it was shown using SECCM‐interference reflectance microscopy that O_2_ and CO_2_ can impact the transport over the liquid/gas interface on the local pH swing during ORR in a mixture of NaHCO_3_ and CaCl_2_ and impact the CaCO_3_ precipitation.^[^ ^42^ ^]^ Compared to ORR, where an increase in the local pH is expected, in OER, we expect a decrease in the local pH, which may trigger different effects on the electrocatalysis.

**Figure 2:**
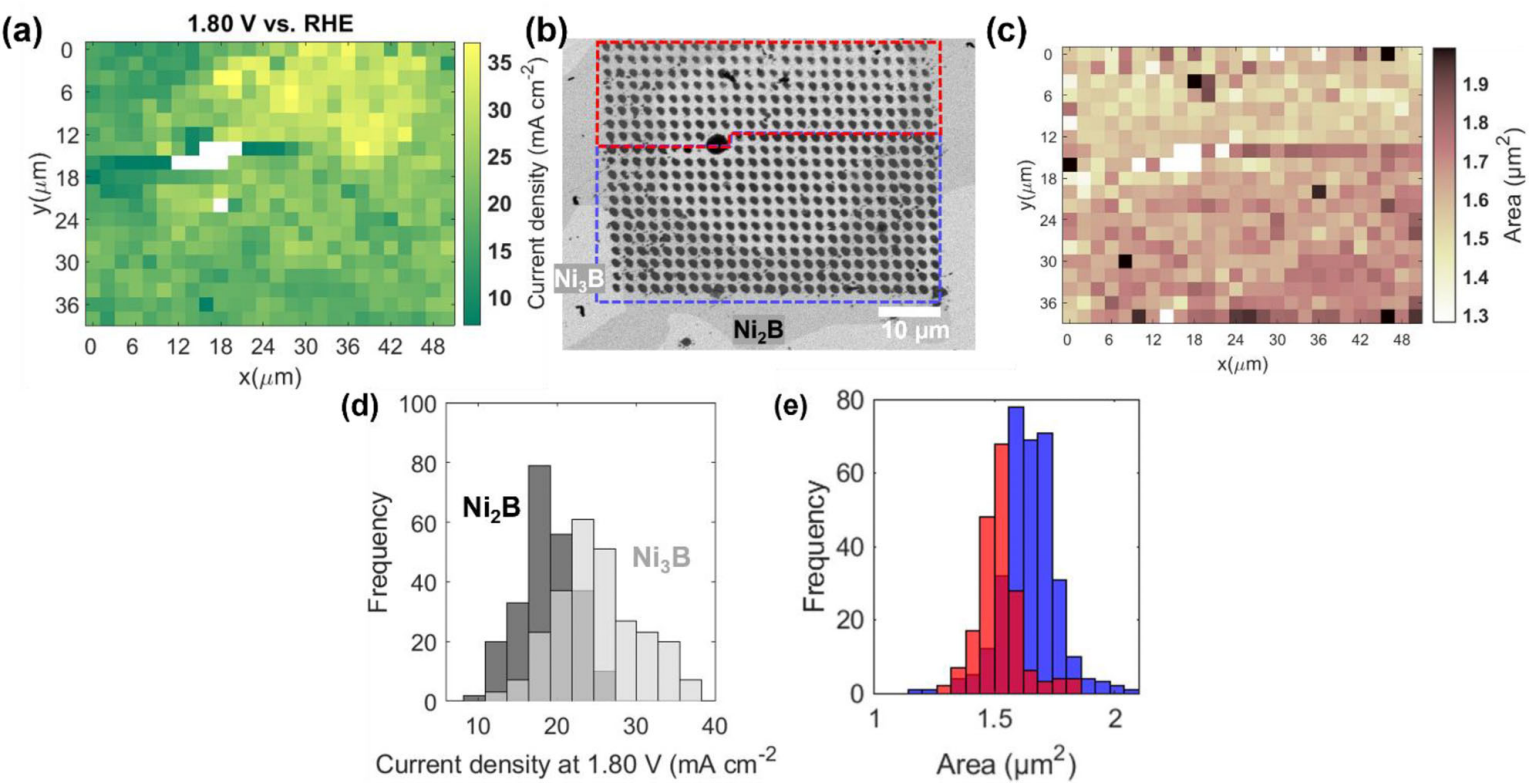
a) Current density map at 1.8 V versus RHE for the SECCM experiment in Ar. b) SEM image of Ni_70_B_30_ showing the phases probed during the SECCM scan and the footprints left after scanning using a nanopipette with a diameter of ≈344 nm (Figure S2b, Supporting Information); the red and blue frames indicate the measured areas before and after we observed a big black dot on the SEM image. c) Area of the footprints of the MAs calculated based on Figure 2b. d) Histogram with the current distributions at 1.8 V versus RHE for the experiments in Ar: the dark gray corresponds to Ni_2_B, while the light gray to the Ni_3_B phase. The intermediate gray color results from the overlap of the dark and light gray. The average current densities are 18.7 ± 0.4 mA cm^−2^ for Ni_2_B and 25.2 ± 0.7 mA cm^−2^ for Ni_3_B and were obtained by analyzing n = 237 MAs on Ni_2_B and n = 259 MAs on Ni_3_B. e) Histogram with the distribution of footprint areas for the different MAs grouped by regions as indicated by the red and blue polygons in the SEM image in (b). red show a maximum of 1.52 ± 0.02 mA cm^−2^ on red and of 1.65 ± 0.02 mA cm^−2^ on blue and were obtained by processing n = 187 (red) and n = 324 (blue) MAs. Data are presented as mean ± 95% confidence interval. The LSVs were recorded in 0.1 M KOH electrolyte at a 1.0 V s^−1^ scan rate.

To deconvolute the factors that may impact the measured OER activity on Ni‐B phases, we conducted a series of controlled experiments in different atmospheres using the environmental chamber (Figure S1, Supporting Information), in which we maintained a continuous flow of a pre‐humidified gas throughout the experiment. In the first stage, we evaluated the Ni_3_B and Ni_2_B OER electrocatalytic activity under exclusion of O_2_ by flushing the cell with a humidified Ar flow (30 mL min^−1^). Interestingly, in Ar, Ni_2_B, and Ni_3_B show a clear difference in their OER electrocatalytic activity, as depicted by the current density map derived at 1.8 V versus RHE (**Figure** **2**a) that was further correlated to the SEM image after the SECCM scan (Figure 2b). The results indicate that the Ni‐rich boride (Ni_3_B) shows a higher OER activity than Ni_2_B, which contradicts previous findings on Ni‐B nanoparticles.^[^ ^25^, ^26^ ^]^ The histogram based on the current densities at 1.8 V versus RHE, grouped by phases, depicted in Figure 2d,e, supports this statement. Under the Ar flow, the distribution of the current densities recorded on the Ni_3_B and Ni_2_B phases is centered at 25.2 ± 0.7 mA cm^−2^ and 18.7 ± 0.4 mA cm^−2^, respectively. Furthermore, we observed that the current densities recorded in the OER region (Figure 2d) were higher than those recorded under stagnant air atmosphere (without gas flow, Figure S4, Supporting Information), o suggesting a significant impact of the gas atmosphere used in the SECCM measurement.

Over the SECCM scan, we observed a substantial dispersion in the estimated current density (Figure 2d). A deeper analysis of the data reveals a change in the SECCM measured areas (MAs) size in line 8 on the SEM image taken after the SECCM scan (Figure 2b), possibly due to a change in the capillary size. While before line 8, the average area of the MAs was 1.52 ± 0.02 µm^2^, this increased to 1.65 ± 0.02 µm^2^ after it (Figure 2c,e). Also, in this case, an inverse correlation is observed between the recorded current densities and the MAs, partly explaining the lower current densities recorded in the lower part of the current map for both phases (Figure S5, Supporting Information). The dominant impact of the footprint size on the increased current densities recorded in the Ar atmosphere (25.2 ± 0.7 mA cm^−2^ compared to 11.6 ± 0.3 mA cm^−2^ in stagnant air on the Ni_3_B) can be excluded, since bigger footprints are observed compared with the measurement conducted in stagnant air, which would induce an opposite trend in the activity. At this point, we hypothesize that different factors can cause the critical difference in measured OER activity of Ni_3_B and Ni_2_B using Ar or air outside the SECCM droplet: i) the electrochemically formed O_2_ is removed faster via diffusion or convection from the surface due to enhanced mass transport facilitated by Ar flow outside the nanodroplet, ii) the presence of CO_2_ in the air, despite in low concentration, influences the electrolyte composition, by forming carbonate/bicarbonate species that may lead to a decrease of the electrolyte pH in the nanodroplet. The effect of the O_2_ transport on the recorded current can be defined by several parameters, including the droplet size (as mentioned above), the concentration of O_2_ outside the droplet, and the presence of gas flow, which introduces a convective component to the mass transfer. Regarding the impact of CO_2_, it has been reported that CO_2_ uptake from air causes the acidification of water droplets (moderate pH decrease by 0.2‐0.4 units).^[^ ^40^ ^]^ This effect is faster in smaller droplets,^[^ ^39^ ^]^ especially in KOH electrolyte compared to more neutral ones, which can be used as an effective CO_2_ absorber.^[^ ^38^ ^]^ So, it is reasonable to expect that the uptake of CO_2_ by the electrolyte nanodroplet during the OER measurement leads to a local pH decrease due to the formation of carbonate and bicarbonate ions, and eventually to carbonate salt crystals.^[^ ^42^ ^]^

**Figure 3:**
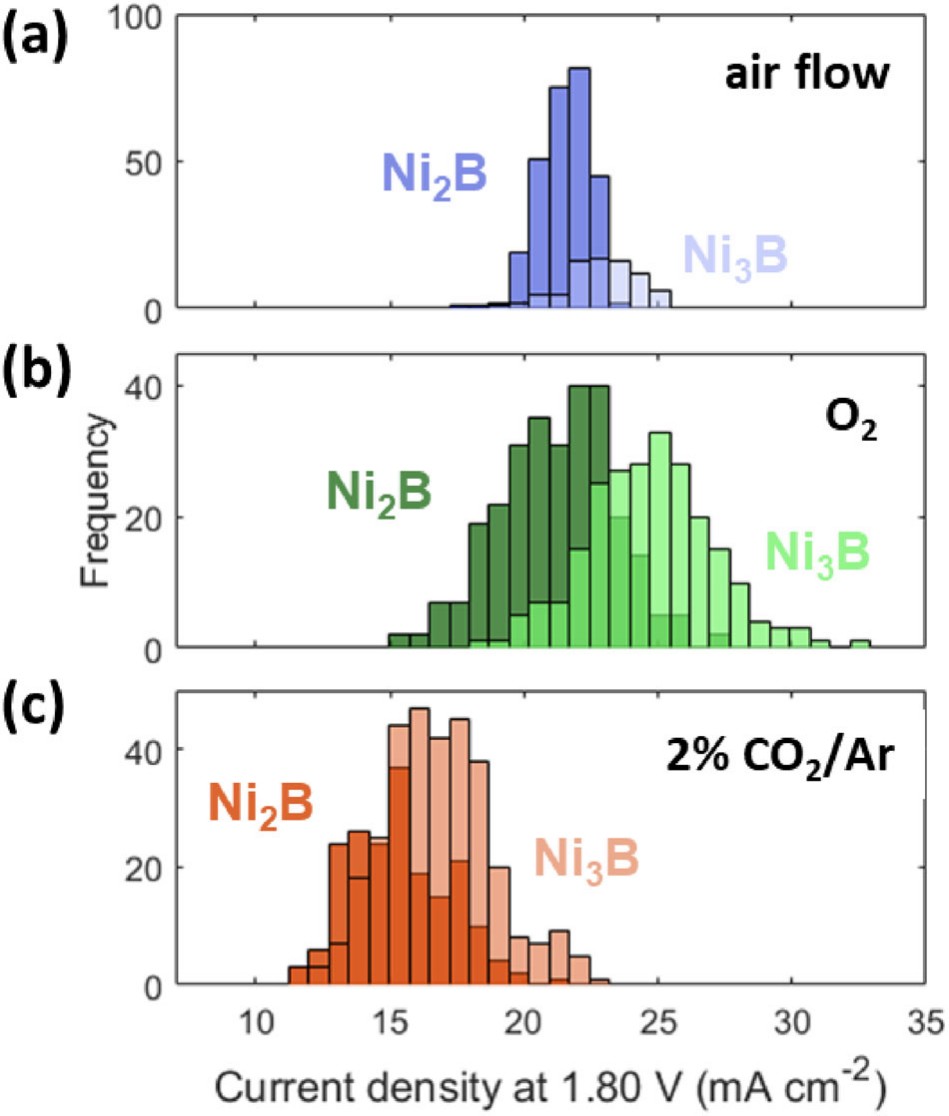
Histograms showing the current density distribution recorded at 1.8 V versus RHE on Ni_3_B and Ni_2_B derived from the SECCM experiments conducted under a 30 mL s^−1^ a) air flow, b) O_2_ flow, and c) 2%CO_2_/Ar flow_._ The darker color depicts the Ni_2_B and the lighter one the Ni_3_B phase, while the intermediate color is due to the overlap of the dark and light color. The average of the current densities calculated in the different atmospheres over different MAs are the following: Air flow:Ni_2_B = 21.5 ± 0.1 mA cm^−2^ (n = 278), Ni_3_B = 22.8 ± 0.3 mA cm^−2^ (n = 80); O_2_:Ni_2_B = 21.2 ± 0.2 mA cm^−2^ (n = 282), Ni_3_B = 24.7 ± 0.3 mA cm^−2^ (n = 234); 2% CO_2_/Ar:Ni_2_B = 15.3 ± 0.2 mA cm^−2^ (n = 192), Ni_3_B = 16.8 ± 0.2 mA cm^−2^ (n = 322). Data are presented as mean ± 95% confidence interval.

To understand the impact of convection on the measured OER activity, additional SECCM experiments in which 30 mL min^−1^ air, O_2_, or CO_2_ diluted in Ar (2% CO_2_/Ar) were conducted. The results clearly confirm that gas convection has a pronounced influence on the recorded current densities (**Figure** **3**a). Only by using an air flow (Figure S6, Supporting Information), we observe an increase in the current densities obtained on both Ni_3_B and Ni_2_B, as well as a slight difference in the OER electrocatalytic activity of the two phases, compared with the stagnant air. The mean current density within the Ni_3_B phase is 22.8 ± 0.3 mA cm^−2^, which is just slightly lower than the 25.2 ± 0.7 mA cm^−2^ recorded under Ar atmosphere. To understand the impact of O_2_ and CO_2_ on the measured OER activity, we performed additional experiments by feeding the chamber with 30 mL min^−1^ of humidified O_2_ or CO_2_ diluted in Ar (ca. 2% CO_2_). Also, in the presence of O_2_ flow, differences in the OER electrocatalytic activity between the two Ni‐B phases are evident (Figure S7, Supporting Information), with Ni_3_B enabling higher OER current densities to be recorded compared to Ni_2_B at potentials higher than 1.7 V versus RHE, which is further supported by the derived histograms for each phase at 1.8 V versus RHE (Figure 3b). Similarly, in the presence of CO_2_, the OER activity of Ni_3_B and Ni_2_B is easily distinguishable from the current density maps at 1.8 V versus RHE (Figure S8, Supporting Information; Figure 3c). The highest increase in current density relative to the stagnant air environment was obtained under Ar flow, whereas the experiment using 2% CO_2_ diluted in Ar presented the smallest increment from the stagnant air environment. The current densities increase in the order: stagnant air < 2% CO_2_/Ar < flowing air < O_2_ ∼ Ar (Figure S9, Supporting Information). This trend is independent on the nature of Ni‐B phase, on which it is measured, namely a similar trend was also observed for the Ni_2_B phase (Figure S10, Supporting Information). To exclude that the observed trend is caused by the different footprints observed on the ingot surface after the SECCM measurement, we performed correlations of the recorded current density with the footprint areas depending on the gas atmosphere (Figure S11, Supporting Information). For the stagnant air and O_2_ flow, a linear negative dependence is observed between the recorded current density and the nanopipette footprint, while in the case of Ar and 2% CO_2_/Ar flows, no linear correlation is noticed, even when analyzing the trends on the Ni_2_B and Ni_3_B phases. While the footprints obtained in the presence of O_2_ are the smallest, with values between 0.5 – 0.8 µm^2^, which may justify the increased current density, in Ar, the biggest footprints are observed (> 1.5 µm^2^). Thus, the footprint size alone cannot justify the OER activity trend. In addition, despite the footprints of the measurement in 2% CO_2_/Ar being similar to those obtained when using stagnant air, the current densities recorded in the presence of CO_2_ do not show the negative linear dependence on the footprint as those in stagnant air or O_2_.

**Figure 4:**
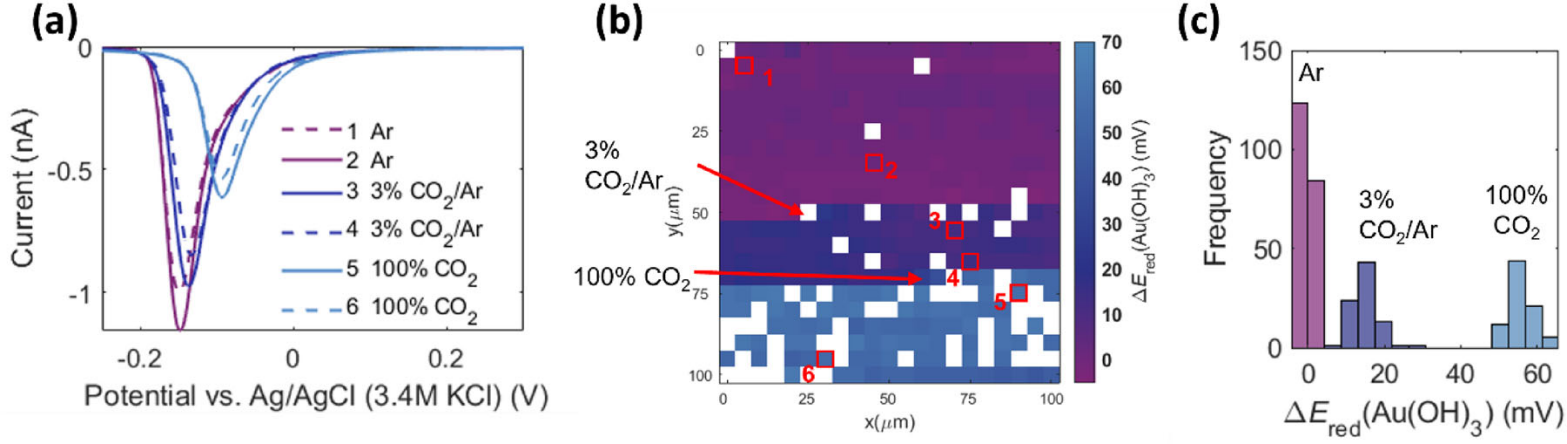
a) Cathodic scan of selected voltammograms recorded with a scan rate 1 V s^−1^ in 0.1 M KOH electrolyte using Au working electrode and a nanopipette of ≈386 nm in diameter. The dashes and solid lines were used for a better visualization of the selected points recorded under the same conditions b) Map and c) histograms, illustrating the Au(OH)_3_ reduction peak potential shift Δ*E*_red_(Au(OH)_3_) induced by the change in the SECCM atmosphere (indicated by the arrows). Ar: 0.0 ± 0.2 mV; 3% CO_2_/Ar: 14.4 ± 0.7 mV; 100% CO_2_: 56.0 ± 0.8 mV. The MAs analyzed are n = 207 (Ar), n = 83 (3% CO_2_/Ar), and n=82 (100% CO_2_). Data are presented as mean ± 95% confidence interval.

At this point, we can conclude that the presence of CO_2_ in the atmosphere surrounding the droplet has one of the highest detrimental impacts on the measured electrocatalytic activity. To justify whether the pH of the 0.1 M KOH electrolyte droplet changes due to the different concentrations of CO_2_ used in the SECCM measurements, we performed an additional experiment using Au as the working electrode, in which the pH‐dependent reduction of Au(OH)_3_ to Au^[^ ^43^, ^44^ ^]^ was monitored. Cyclic voltammograms were recorded from ‐0.3 up to 1.5 V versus Ag/AgCl/3.4 M KCl (scan rate: 1 V s^−1^). Initially, an Ar flow was used, followed by a stepwise increase of the CO_2_ concentration to 3% CO_2_ and subsequently to 100%. The Au(OH)_3_ reduction peak potential displayed an anodic shift when CO_2_ was introduced in the atmosphere during the SECCM experiment, and the magnitude of this shift increased with the CO_2_ concentration (**Figure** **4**a). The difference in the peak potential with respect to the average peak potential recorded on MAs in the presence of Ar flow (first 210 points) is depicted in Figure 4b. Three regions are identified, correlating with the concentration of CO_2_ in the used stream. The average peak shift was 15 mV when 3% CO_2_/Ar was used, while 56 mV shift was observed in 100% CO_2_ (Figure 4c). Remarkably, the shifting was similar for all points in each region, and any further increase was identified for the later spots of each zone. It suggests that the droplet is formed for each spot and reaches a similar stationary state after the elapsed time between two consecutive measurements (≈10 s).

According to previous reports, the Au(OH)_3_ reduction peak exhibited a Nernstian behavior, and the shift in the potential is proportional to the shift in the pH with a slope of 58 mV (Δ*E* = 58·ΔpH), which is close to the 59 mV expected according to the Nernst equation.^[^ ^45^, ^46^ ^]^ It can be expected that the relation holds true in the SECCM experiments, according to which a 15 mV shift in the potential will correspond to a pH decrement of ≈0.26 units for 3% CO_2_/Ar. A 56 mV potential shift in an experiment with 100% CO_2_ corresponds to a decrease of 0.97 pH units. Considering the shift in the pH to correct the potential scale versus RHE, the “real” current density at 1.8 V versus RHE for the experiments using 3% CO_2_ in Ar would be ≈21.7 mA cm^−2^ for Ni_3_B, which is closer to that recorded under an Ar atmosphere. Thus, the low activity recorded on both Ni_2_B and Ni_3_B when CO_2_ is present in the gas atmosphere can be partially explained by an error in the calculation of the *E* versus RHE due to changes in the pH of the electrolyte. Still, the magnitude of the change can be different during OER experiments, when a local increase in proton concentration may take place. The findings highlight that in an SECCM measurement conducted in alkaline media at the nanoscale, CO_2_ can have a significant impact despite its low concentration in the air, and, therefore, needs to be excluded when evaluating the “intrinsic” activity of the electrocatalyst.

**Figure 5:**
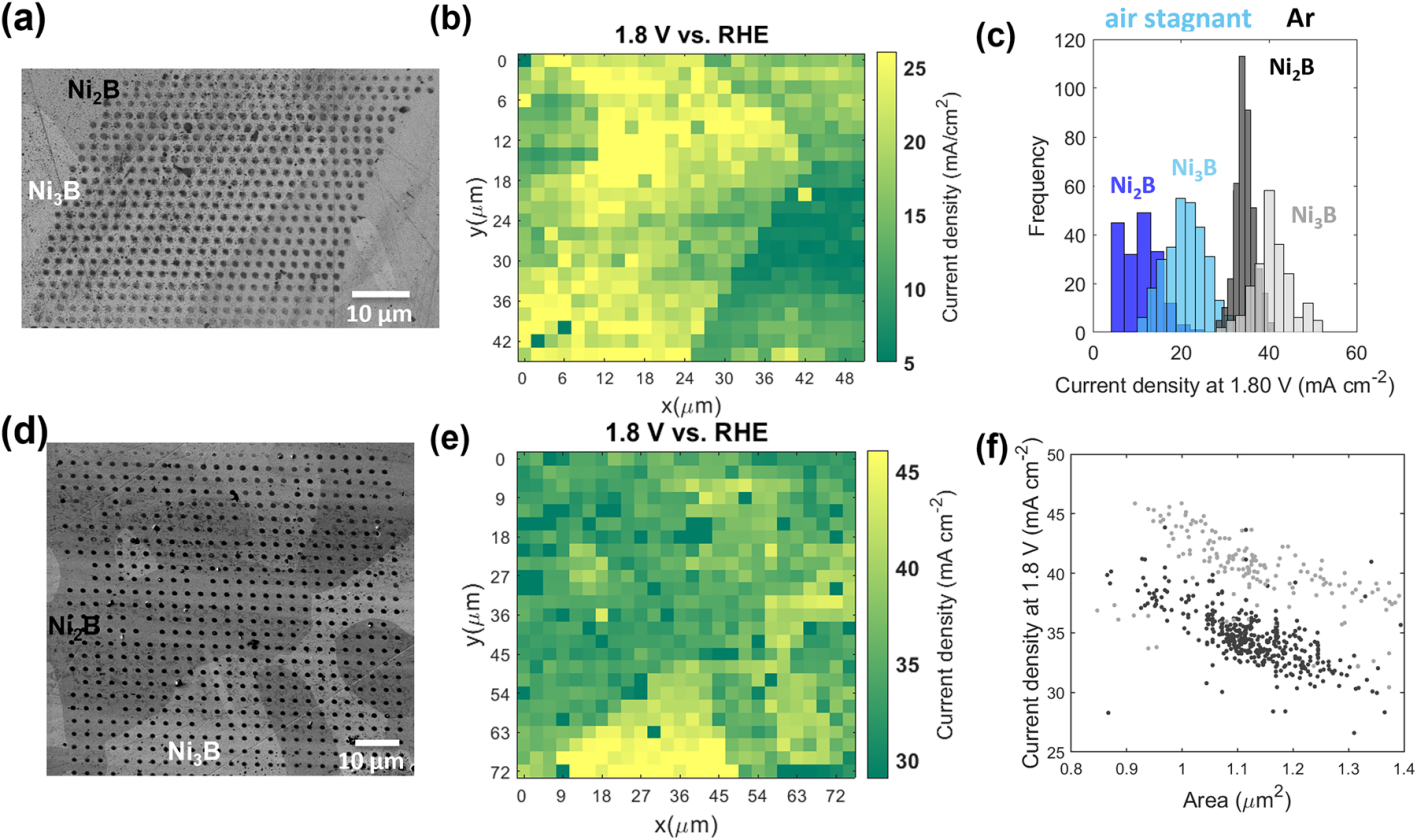
SEM images of the SECCM scans conducted on a freshly polished Ni_70_B_30_ surface showing the phases present and the footprints left after scanning in a) stagnant air and d) Ar flow; Current density maps at 1.8 V versus RHE for the experiment performed in b) stagnant air and e) Ar flow; c) Histograms showing the current densities distribution recorded at 1.8 V versus RHE grouped by atmosphere (stagnant air ‐ in blue, Ar flow ‐ in gray) and Ni‐B phases (Ni_2_B‐ dark color, Ni_3_B‐ light color) according to the SEM image; stagnant air: Ni_2_B = 10.6 ± 0.6 mA cm^−2^; Ni_3_B = 21.0 ± 0.5 mA cm^−2^. Ar: Ni_2_B = 34.5 ± 0.2 mA cm^−2^; Ni_3_B = 40.7 ± 0.6 mA cm^−2^. The MAs analyzed are n = 175 for Ni_2_B and n = 291 for Ni_3_B in stagnant air and n = 399 for Ni_2_B and n = 202 for Ni_3_B in Ar. f) Current density versus footprint area correlation derived for the Ni_2_B (dark gray) and Ni_3_B (light gray) recorded under Ar flow. Data are presented as mean ± 95% confidence interval.

In the last step, we investigated the OER activity of the Ni_2_B and Ni_3_B phases of the freshly polished Ni_70_B_30_ ingot under stagnant air (**Figure** **5**; Figure S12, Supporting Information) and Ar flow, the SECCM conditions in which the lowest and the highest OER current densities were previously recorded. On a freshly polished surface, higher current densities are recorded in both atmospheres. The increment observed under Ar flow can be partly explained by the smaller footprints left after the SECCM experiment, which range between 0.8 and 1.4 µm^2^ (see Figure 5f). Current densities of ≈30 and ≈38 mA cm^−2^ are obtained on Ni_2_B and Ni_3_B, at ≈1.4 µm^2^ footprint, which are higher than those initially obtained (Figure 2; Figure S5, Supporting Information). This may indicate changes in the catalyst surface of non‐noble metal catalysts during storage, which can impact their electrocatalytic activity. Interestingly, on a freshly polished Ni_70_B_30_ surface, the difference between the Ni_3_B and Ni_2_B OER activity becomes evident also in stagnant air (Figure 5a,b). The average current density measured under Ar within Ni_3_B phase (40.7 ± 0.3 mA cm^−2^) is approximately two times higher than that measured in stagnant air (21.2 ± 0.2 mA cm^−2^) (Figure 5c) and confirms that Ni_3_B is more active for OER than Ni_2_B under both stagnant air and Ar flow, as shown in the current density maps and the corresponding SEM images (Figure 5). It is important to note that in these experiments the footprint areas obtained under air and Ar were comparable (Figure S12a–d, Supporting Information), enabling the comparison of the two phases under similar conditions.

These results indicate that the influence of the environment surrounding the droplet in SECCM onto the recorded OER currents is very complex in nature. Especially, the presence of CO_2_ adds complexity, most probably due to the established equilibrium in 0.1 M KOH solution between CO_2_, HCO_3_^−^ and CO_3_^2−^ species. Nevertheless, all present results clearly reveal that Ni_3_B is more active than Ni_2_B, a result that strongly contrasts with earlier work studying the effect of the composition, where Ni_2_B was identified as being more OER‐active compared to Ni_3_B.^[^ ^25^, ^26^ ^]^ However, the previous studies dealt with highly amorphous phases that underwent oxidation via consecutive cycling and/or storage. In the present study, we show that by performing SECCM measurements of a well‐characterized crystalline sample, containing both phases simultaneously, we enabled measuring OER activities under well‐controlled.

## 3 Conclusion

In the present study, the OER electrocatalytic activity of Ni_2_B and Ni_3_B simultaneously present in a Ni_70_B_30_ ingot, was assessed through controlled electrochemical measurements using SECCM, revealing that Ni_3_B is more active than Ni_2_B. By controlling the atmosphere surrounding the hanging nanodroplet, we probed the activity of the Ni_2_B and Ni_3_B in one single experiment, ensuring high reproducibility between the experimental conditions used to distinguish the OER activity of two OER‐active Ni‐B phases. In addition, we provide important experimental insights that will enable higher OER currents to be recorded, with decreased impact of mass transport processes, in SECCM. Properties such as local pH and the mass transport at the gas‐liquid interface are affected by the chemical nature of the gas atmosphere as well as the presence or absence of a gas convection outside of the droplet. The use of an Ar gas flow, as well as the exclusion of CO_2_ from the atmosphere, are found to be key factors in enabling increased current densities to be recorded on both Ni_2_B and Ni_3_B. The atmosphere control during SECCM experiments allowed to distinguish the intrinsic OER activity for two Ni‐B compounds and confidently state that the crystalline Ni_3_B phase shows higher OER current densities compared to those measured for Ni_2_B phase. Importantly, the magnitude of the difference between the two phases depends strongly on the aforementioned experimental conditions. The present study not only provides valuable insight into the intrinsic OER activity of Ni‐B intermetallic compounds but also addresses important experimental aspects that need to be integrated in the OER measurement protocols to enable the evaluation of electrocatalysts at the nanoscale using SECCM method.

## Conflict of Interest

The authors declare no conflict of interest.

## Supporting information



Supporting Information

## Data Availability

The data that support the findings of this study are openly available at Zenodo at https://doi.org/10.5281/zenodo.17769623
