# Supplementary material for: Impact of the Gas Atmosphere at the Triple Boundary Phase on the Measured Oxygen Evolution Reaction Activity of Ni2B/Ni3B Electrocatalysts
Source: Small Methods. 2025 Dec 4;10(3):e01880. doi: 10.1002/smtd.202501880 (PMC12893308; doi:10.1002/smtd.202501880)
Supplement: Supplementary file 1 — Supporting Information [file SMTD-10-e01880-s001.docx]

**Impact of the Gas Atmosphere at the Triple Boundary Phase on the Measured Oxygen Evolution Reaction Activity of Ni_2_B/Ni_3_B Electrocatalysts**

Lithin Madayan-Banatheth^[a]§^, Alejandro E. Perez-Mendoza^[a]§^, Ulrich Burkhardt^[b]^, Iryna Antonyshyn*^[b,c]^, Corina Andronescu*^[a,d]^

[a] L. Madayan-Banatheth, Dr. A. E. Perez-Mendoza, Prof. Dr. C. Andronescu
Chemical Technology III
University of Duisburg-Essen
Carl-Benz-Straße 199, 47057 Duisburg, Germany
E-mail: corina.andronescu@uni-due.de

[b] Dr. U. Burkhardt, Dr. I. Antonyshyn
Max-Planck-Institut für Chemische Physik fester Stoffe
Nöthnitzer Straße 40, 01187 Dresden, Germany
E-Mail: iryna.antonyshyn@cpfs.mpg.de

[c] Dr. I. Antonyshyn
Fritz-Haber-Institut der Max-Planck-Gesellschaft
Faradayweg 4-6, 14195 Berlin, Germany

[d] Prof. Dr. C. Andronescu
CENIDE, Center for Nanointegration, University of Duisburg-Essen
Carl-Benz-Straße 199, 47057 Duisburg, Germany

[^§^] These authors contributed equally to this work.

S1. Experimental Section

S1.1 Synthesis and characterization of Ni_2_B/Ni_3_B electrocatalysts

The pieces of crystalline boron (Alfa Aesar, 99.999 %) were weighed and wrapped into 0.025 mm thick Ni foil (Alfa Aesar, 99.99% metal basis). To reach the atomic ratio of Ni:B as 7:3 and avoid two volumetric specimens for melting, Ni slugs (Alfa Aesar, Puratronic, 99.995 % metal basis) were added. The weighted mixture of Ni and B was melted using arc melter with a water-cooled copper bottom under a partial pressure of argon. Melting was repeated a few times to achieve the sample homogeneity and complete the reaction. Finally, the obtained ingot was placed in ZrO_2_ crucible, which was sealed into a Ta container in the argon box. This container was sealed into a quartz tube, which was placed into the resistance furnace at 1000 ^o^C for 16 days. After homogenization treatment, the ampoule was quenched in cold water.

The obtained Ni_70_B_30_ ingot was cut using a wire saw in the form of discs (ca. 8 mm in diameter), which were further polished and used for detailed bulk characterization and electrochemical experiments.  The polishing was carried out by rotating the plate with SiC grinding papers (Grit 800, 1200, 2400 and 4000) on a grinding machine (LaboPol-21) at 250 rpm. Further polishing to mirror surface was performed with diamond solutions (diamond particle size 3,1, and ¼ μm in diameter) and water-based green lubricant from Struers on a rotating MD Dur plate at 800 rpm using a polishing machine (RotoPol-15).

For bulk characterization of Ni_70_B_30_ specimen, powder X-ray diffraction (PXRD), light microscopy and scanning electron microscopy accompanied by wavelength-dispersive X-ray spectroscopy were employed. For PXRD, the pieces of the synthesized ingot were ground in an agate mortar and put between two Kapton foils onto a special PXRD holder. X-ray powder diffraction patterns were measured in transmission geometry with a Huber Imaging Plate Guinier Camera G670 (Cu *K*α_1_, *λ* = 1.54059 Å). The phase analysis was performed by comparing the experimental patterns with calculated ones using the program WinXPOW.^[1]^ For lattice parameter determination, the internal standard LaB_6_ (a = 4.1569 Å) was added to the samples before PXRD. The indexing and lattice parameters determination were realized via implication of software package WinCSD.^[2]^ The homogeneity of Ni_70_B_30_ specimen was examined by light microscopy (LM-Zeiss Axioplan 2 light microscope, CCD camera and Olympus stream software^[3]^) and scanning electron microscopy SEM (JEOL JSM-7800F microscope). The compositions of the observed Ni-B phases were determined via wavelength-dispersive X-ray spectroscopy (WDXS, CAMECA electron microprobe SX100 setup, W cathode, Ni_3_B was used as reference for Ni and B quantification). The PAP matrix correction mode^[4]^ was used for chemical composition calculations.

**S1.2 Scanning Electrochemical Cell Microscopy (SECCM)**

Electrochemical measurements were performed in a home-built scanning electrochemical cell microscopy (SECCM), the experiments were controlled using an FPGA card (USB-7855R) and a modified LabVIEW (National Instruments) software based on the Warwick Electrochemical Scanning Probe Microscopy (WEC-SPM) toolbox.^[5, 6]^ The sample holder was coarsely moved by x,y,z-stepper motors (Owis) with a LStep PCIe (Lang) controller to choose a particular coarse region of interest on the surface and approach the surface to the SECCM tip to a distance of around 50 μm with the aid of a camera (Motic) equipped with VZM^TM^ 200i zoom lenses (Edmund Optics) and a light source (EUROMEX cold light source LED 2-Spot). The surface was scanned in a hopping mode using a x,y,z-piezo cube (C3.100, PIEZOCONCEPT) with its respective controller, which finely moved the pipette holder.

The electrochemical cell is formed in each spot upon contact of the hanging droplet of the tip and the surface of Ni_70_B_30_ sample. The surface contacted by the electrolyte acts as a working electrode, while a leakless Ag/AgCl/3.4 M KCl (Innovative Instruments) inserted inside the single-barrel quartz pipette filled with 0.1 M KOH electrolyte was used as reference electrode/counter electrode. The diameters of the openings of the nanopipetts are summarized in Table S2. The voltage was applied to the system using the FPGA card and the current flowing through the system was measured using a variable gain transimpedance amplifier (DLPCA-200, FEMTO Messtechnik). The amplifier, the camera, the sample, and the pipette holders were installed on a vibration-damping table (Vision IsoStation, Newport), which is inside a copper mesh Faraday cage (VIS-FDC-3648, Newport). As illustrated in Figure S1, the sample was placed inside a custom-made Acrylonitrile Butadiene Styrene chamber, which allows gas to be introduced from the side. The chamber is equipped with a transparent polymethylmethacrylate lid featuring a 1 cm diameter circular hole to enable the positioning and movement of the nanopipette. Gas flow is regulated using mass flow controllers (EL-Flow select F-201CV-100, Bronkhorst Instruments GmbH), the flows are reported as volumetric flow (mL min^-1^) using the European standard reference conditions for the conversion (temperature of 20°C, and a pressure of 1 atm). Before entering the chamber, the supplied gases are passed through a water column for humidification.


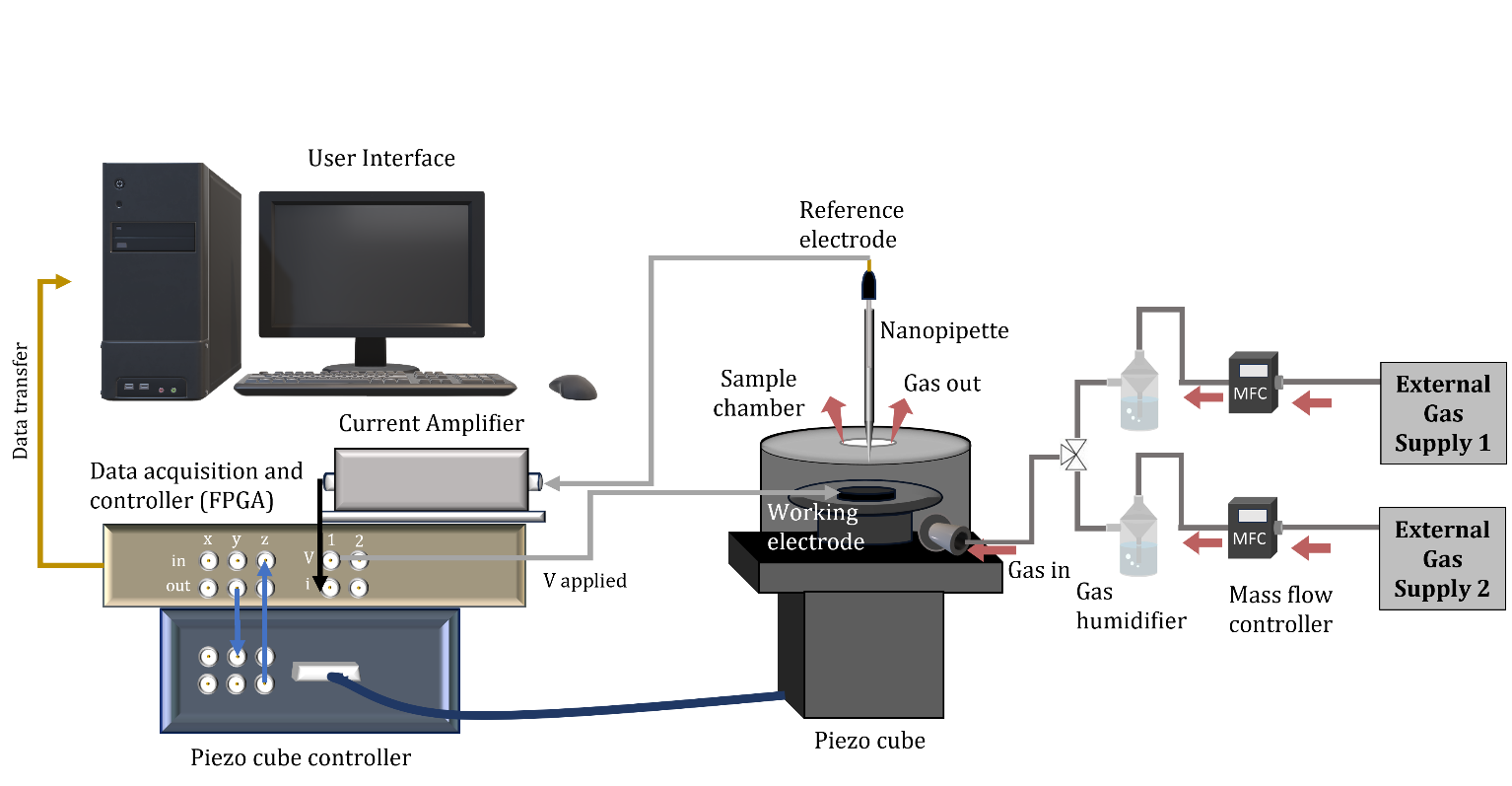


**Figure S1.** Schematic illustration of the setup used for SECCM measurements under controlled atmosphere. Two external gas supplies are independently regulated using mass flow controllers (MFC), then bubbled through separate water columns (gas humidifiers) before being directed into the experimental chamber. The chamber houses the working electrode and an opening at the top to allow the nanopipette to approach the sample surface and conduct measurements. The setup allows precise control over gas composition at the sample surface.

*Electrochemical parameters*

The electrolyte used for all electrochemical and SECCM measurements was 0.1 M KOH, prepared from 87.8% purity KOH pellets (Merck) and Milli-Q water. The KOH electrolyte used in the experiments was purified to remove Fe impurities using a Chelex 100 cation exchange resin (Sigma-Aldrich, 50-100 mesh). Prior to each experiment, the prepared electrolyte was purged with Ar for at least 30 minutes to remove dissolved oxygen. The deaerated solution was then transferred to the nanopipette and brought to the selected chamber atmosphere (Ar, O_2_, or CO_2_/Ar) before initiating SECCM measurements. The total gas flow rate in each controlled atmosphere experiment was maintained at 30 mL min^-1^ to ensure consistent mass transport and humidity conditions. The potential of the reference electrode was checked before and after electrochemical experiments to adjust the reference scale and discard any significant potential drifting during the experiment. Potentials are converted to the RHE scale by using the equation:

$$E_{\text{RHE }}\text{[V] = }E_{\text{vs. }\text{Ag/AgCl}\text{/3.4 KCl}}\text{ + 0.210 + }E_{\text{OCP }}\text{+ 0.0592 × pH}$$

Where $E_{\text{vs. }\text{Ag/AgCl}\text{ 3.4 KCl}}$ is the applied potential versus the quasi-reference counter electrode (QRCE), 0.210 V is the standard potential of the Ag/AgCl/3 M KCl reference electrode at 25 °C, and $E_{\text{OCP }}$ is the measured open circuit potential difference between the QRCE and the Ag/AgCl/3 M KCl reference electrode. All potential values mentioned are in the RHE scale unless stated otherwise. All cyclic voltammetry (CV) measurements were performed at a scan rate of 1.0 V s^-1^.

**Table S1.** Summary of conditions used for SECCM measurements on Ni_70_B_30_ and Au film electrodes. All potentials are referenced to RHE scale. The potential is expressed vs. RHE.

| **Working electrode** | **Gas** | **Gas flow (mL/s)** | **Number of CV** | **First potential (V)** | **End potential (V)** | **Scan rate**  **(V/s)** |
| --- | --- | --- | --- | --- | --- | --- |
| Ni_70_B_30_ | air stagnant | 30 | 1 | 0.98 | 1.91 | 1 |
| Ni_70_B_30_ | Ar | 30 | 1 | 0.97 | 1.90 | 1 |
| Ni_70_B_30_ | air flow | 30 | 1 | 1.01 | 1.94 | 1 |
| Ni_70_B_30_ | O_2_ | 30 | 1 | 1.00 | 1.93 | 1 |
| Ni_70_B_30_ | 2% CO_2_/Ar | 30 | 1 | 0.99 | 1.92 | 1 |
| Ni_70_B_30__repolished | air stagnant | 30 | 1 | 1.00 | 1.90 | 1 |
| Ni_70_B_30__repolished | Ar | 30 | 1 | 1.00 | 1.90 | 1 |
| Au film | Ar → 3% CO_2_/Ar → CO_2_ | 30 | 2 | 0.73 | 2.53 | 1 |

**S1.3 Pipette fabrication**

Single-barrel pipettes were fabricated by pulling single-barrel quartz glass capillaries (QF120-90-10, Sutter Instruments) using a CO_2_-laser puller (P-2000, Sutter Instruments). The used quartz capillaries were carefully cleaned with wipes soaked in ethanol. The diameter of the tips was measured from the SEM images before usage (Figure S2). Nanopipettes were pulled using a two line program with tuned parameters to get pipettes around 400 nm. Those parameters are: heat (HEAT, controls the power of the laser beam), filament (FIL, controls the scanning length of the laser beam), velocity (VEL, controls the movement of both ends of the capillary before the hard pulling), delay (DEL, controls the time waited after heating and before the hard pulling), and pull (PUL, control the force of the hard pulling).

| Line 1 | HEAT 780 | FIL 4 | VEL 45 | DEL 130 | PUL 0 |
| --- | --- | --- | --- | --- | --- |
| Line 2 | HEAT 780 | FIL 4 | VEL 45 | DEL 130 | PUL 90 |

The “average diameter” (*d*) of the pipette was calculated as the geometric mean of the semi-major (*a*) and semi-minor (*b*) axes, which is equivalent to finding the radius of a circle with the same area as the ellipse ($d=\sqrt{a\cdot b}$). The results of the estimated diameters are presented in the Table S2.

**Table S2.** Estimated average diameters of the pipettes used for the SECCM measurements.

|  | ***a* (nm)** | ***b* (nm)** | ***d* (nm)** |
| --- | --- | --- | --- |
| (a) air stagnant | 566 | 615 | 590 |
| (b) Ar | 421 | 281 | 344 |
| (c) O_2_ | 461 | 336 | 394 |
| (d) 2% CO_2_/Ar | 320 | 489 | 396 |
| (e) air flow | 376 | 537 | 449 |
| (f) varying %CO_2_* | 429 | 347 | 386 |
| (g) air stagnant** | 505 | 493 | 499 |
| (h) Ar** | 568 | 405 | 480 |

*on Au electrode

**on freshly polished Ni_3_B/Ni_2_B specimen


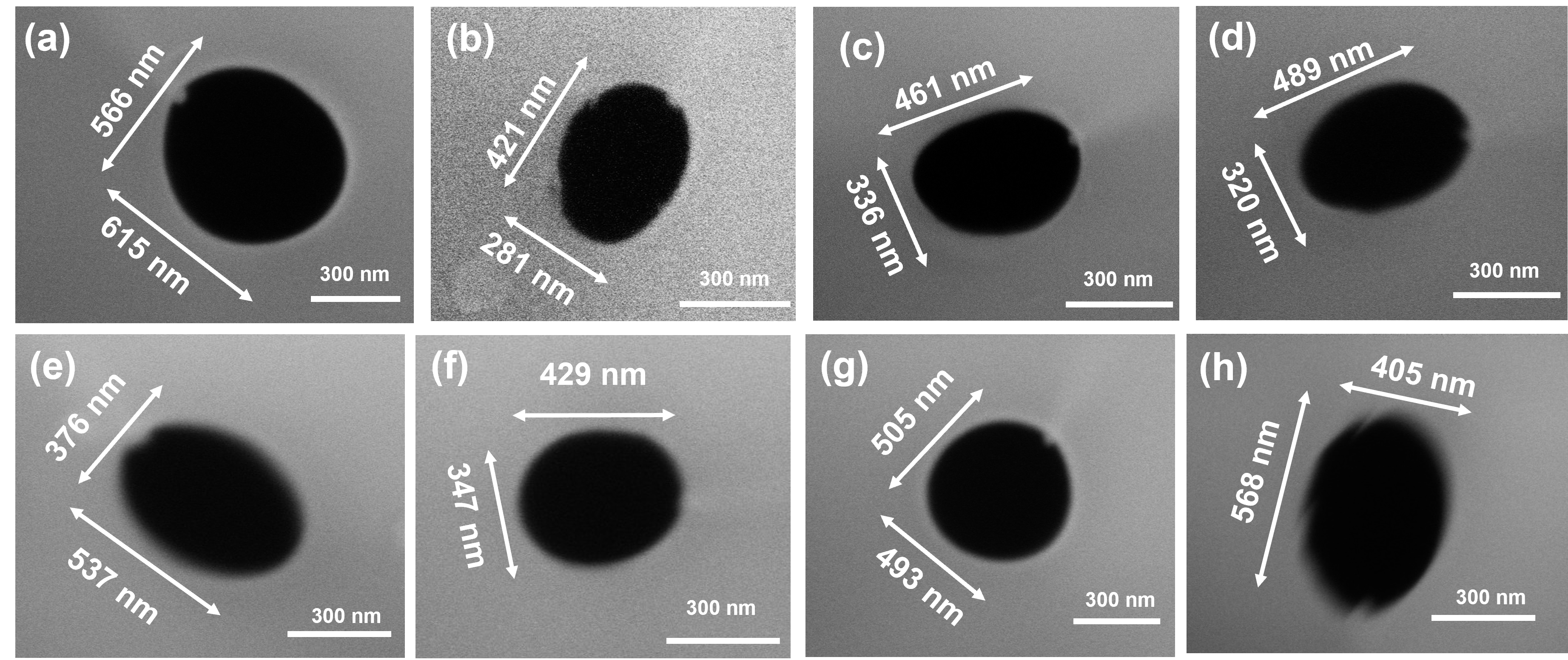


**Figure S2.** SEM images of the tip opening of the pulled single barrel quartz pipettes used for the OER measurements conducted in 0.1 M KOH using different environments: (a) stagnant air, (b) Ar, (c) O_2,_ (d) 2% CO_2_ in Ar, (e) air flow, and (f) experiments on Au under CO_2_/Ar (different concentrations). The pipettes from (g) and (h) were used for the measurements performed on the freshly polished Ni_70_B_30_ surface under stagnant air and Ar, respectively.

**S1.4 Processing SEM images**

The SEM images of KOH footprints left on the surface after the SECCM scan is further processed using the ImageJ software to calculate the area of the footprints and assign them to the respective Ni-B phase on which they are present. The image pixel to distance (in µm) conversion factor obtained from the SEM image is given as input to the ImageJ software, which allows the results obtained from ImageJ to represent the actual length in µm. The extraction of information from the footprints on the SEM image is automated and consists of two steps, as shown in Figure S3. The first step is binarizing the image to black and white using the Auto Local Threshold tool, which sets the KOH footprints and other features with relatively darker contrast to the color white. In the next step, only the footprints are selected using the Analyze Particles tool, which also extracts the surface area and X-Y coordinates of the footprints.

Auto Local Threshold tool computes the threshold for binarizing each pixel according to their intensity within a window of radius r (in pixels) around it. It allows to identify better the features on a background with complex intensity distributions or significant variation in lighting, like in this case, that the background presents regions with light and dark contrast. Ni black thresholding method was used to process the images in this work. The Ni black thresholding method is described as:

pixel = ( pixel > mean + k * standard_deviation - c) ? if yes, the object is the background

There are two parameters: parameter 1 (k value) and parameter 2 (c value) that influence the binarizing algorithm. Throughout this work, the parameters 1 and 2 are kept constant and given the values -0.2 and 0 to select the darker pixels.

The Analyze Particle tool was used to select the KOH footprints from the segmented image, setting the size parameters and circularity. The objects identified with the thresholding process were further filtered to choose just the circular footprints within the expected area size. This tool also allows for calculating the area for given X, Y positions, which is subsequently exported to a comma-separated values file (*.csv). Additionally, polygons enclosing the Ni_3_B phase (distinguished as the light contrast region) present in the image are drawn and the list of coordinates of each polygon are exported as *.csv files. All the files generated are further processed using MATLAB to extract the area and assign it to the corresponding measurement area object, and to identify which spots are positioned inside the Ni_3_B phase. This information enabled the normalization of the currents of each measurement area and their grouping by phase.

The classification of each SECCM footprint within the corresponding area was based on a geometric comparison between the footprint boundaries and the polygon enclosing the Ni₃B phase. Each SECCM landing was represented as a circular footprint of radius *r*, estimated from the geometric area of the droplet, and centered at coordinates (*x*, *y*) obtained from SEM image processing. The four boundary points of this circle—(*x* + *r*, *y*), (*x* − *r*, *y*), (*x*, *y* + *r*), and (*x*, *y* − *r*)—were then compared with the coordinates of the polygons defining the Ni₃B region, as determined from the SEM-derived phase segmentation. If the majority of these boundary points lay within the polygon enclosing the Ni₃B phase, the corresponding measurement was assigned to that phase.

**
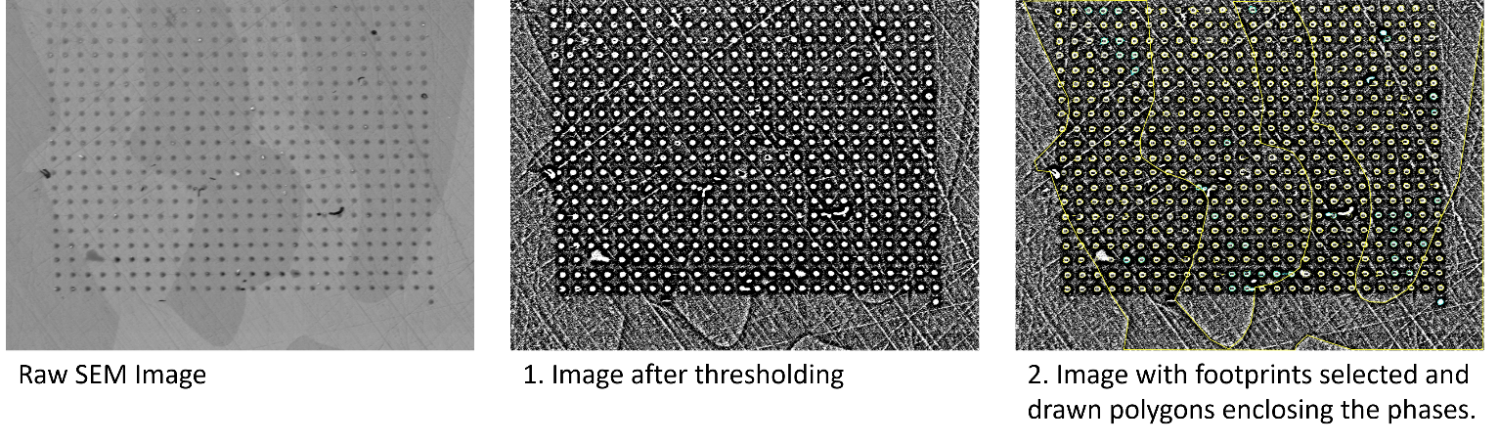
Figure S3.** Example of the SEM image and its processing workflow, including the thresholding and selection of footprints and phases.

**S1.5** **Processing and reporting SECCM data**

The raw *.tdms files obtained after SECCM scans are entirely processed using a set of scripts run on MATLAB software. The scripts enable the extraction of all recorded data into a structure-type variable, followed by the separation based on the spot from which they were collected, with the data being stored in a cell array. Each entry of the array corresponds to one spot, and these data are used to calculate the quantities reported in the text. The potential is converted from Ag/AgCl (3.4 M KCl) scale to RHE scale, and the current is normalized with the footprint area to obtain current density in mA cm^-2^.

*Statistical Analysis*

All statistical analyses, data visualization, and normalization procedures were performed in MATLAB (MathWorks, USA) using built-in statistical functions and custom scripts. Outliers were excluded prior to statistical evaluation using a mean-based criterion, where data points deviating by more than three standard deviations from the group mean were considered outliers. These values were omitted from all subsequent calculations. The statistical parameters reported, including mean values, confidence intervals, and hypothesis tests were therefore based solely on the filtered datasets.

For each measurement, the mean current density ($\bar{\boldsymbol{x}}$) and the 95% confidence interval (= 1.96 × Standard Error of the Mean). The number of valid MAs analyzed (n) for each phase (Ni_2_B and Ni_3_B) for each gas atmosphere is provided in the figure captions. The statistical significance of the difference between current density distributions of Ni_2_B and Ni_3_B phases of each measurement was evaluated using a two sample student’s t-test. This was under the assumption that the current-density data of both phases are independent random normal distributions with equal means and equal but unknown variances. The confidence level 95% corresponds to the significance level (α) of 0.05 used.

**S2. Experimental Results**

**S2.1 Non-controlled atmosphere (stagnant air)**


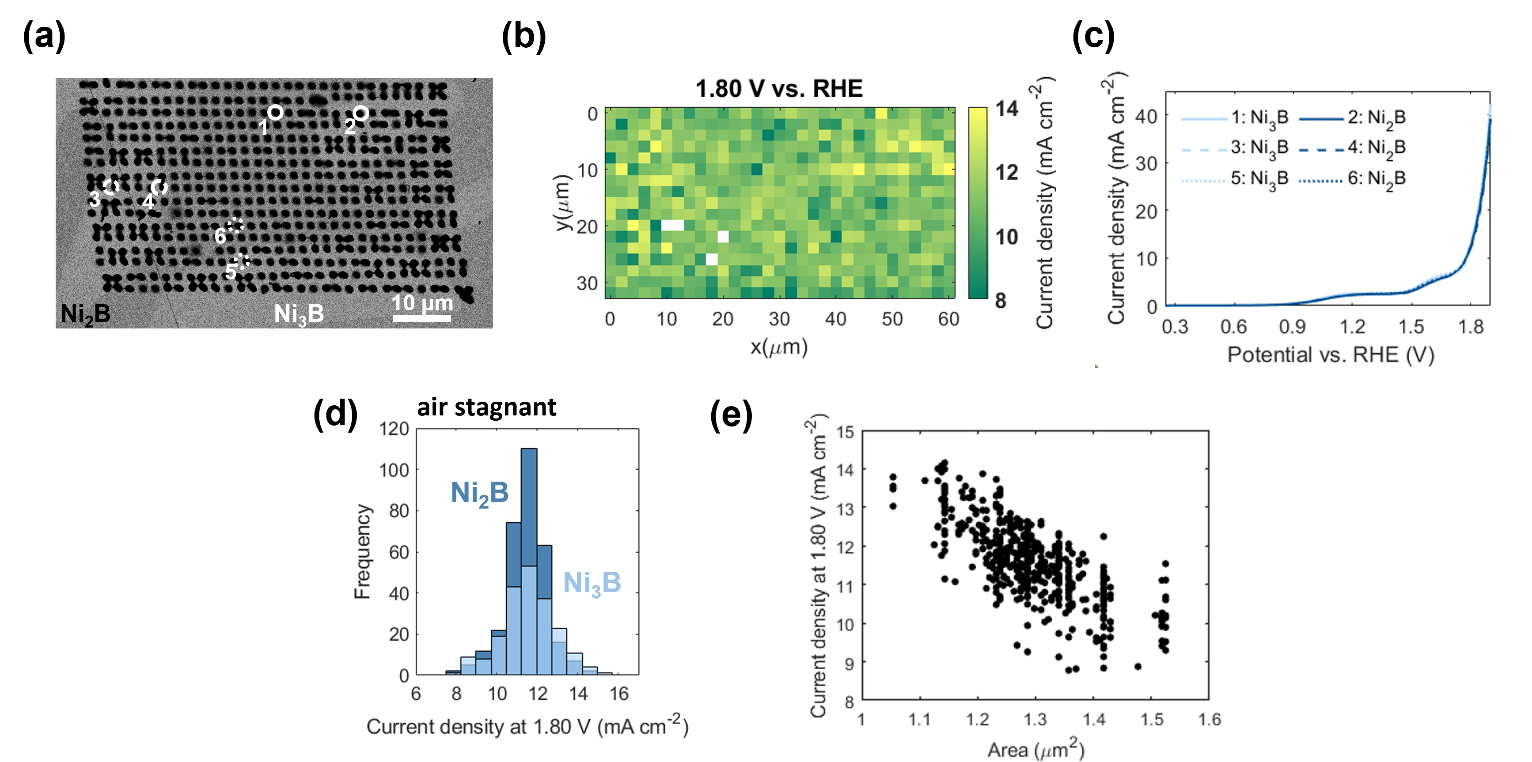


**Figure S4.** (a) SEM image of Ni_70_B_30_ surface with the footprints of the SECCM measurement performed in air using a nanopipette of ~ 590 nm in diameter and a 2 µm hopping distance. (b) Electrochemical activity map derived from the LSVs at 1.8 V vs. RHE. (c) Polarization curves (0.1 M KOH, scan rate of 1 V s^-1^) recorded on several spots of Ni_2_B (dark blue) and Ni_3_B (light blue) marked in (a). (d) Histograms showing the distribution of current densities recorded at 1.8 V vs. RHE grouped by phases according to the SEM image in (a), light blue – Ni_3_B, dark blue – Ni_2_B phase. Ni_2_B: 11.5 ± 0.1 mA cm⁻²; Ni_3_B: 11.6 ± 0.3 mA cm⁻². MAs analyzed on Ni_2_B (n = 313) and Ni_3_B (n = 209). (e) Scatter plot of the current density recorded at 1.8 V vs. RHE as a function of the footprint area of the MAs, based on the processing of the SEM image using ImageJ. Data are presented as mean ± 95 % confidence interval.

**S2.2 Ar atmosphere**


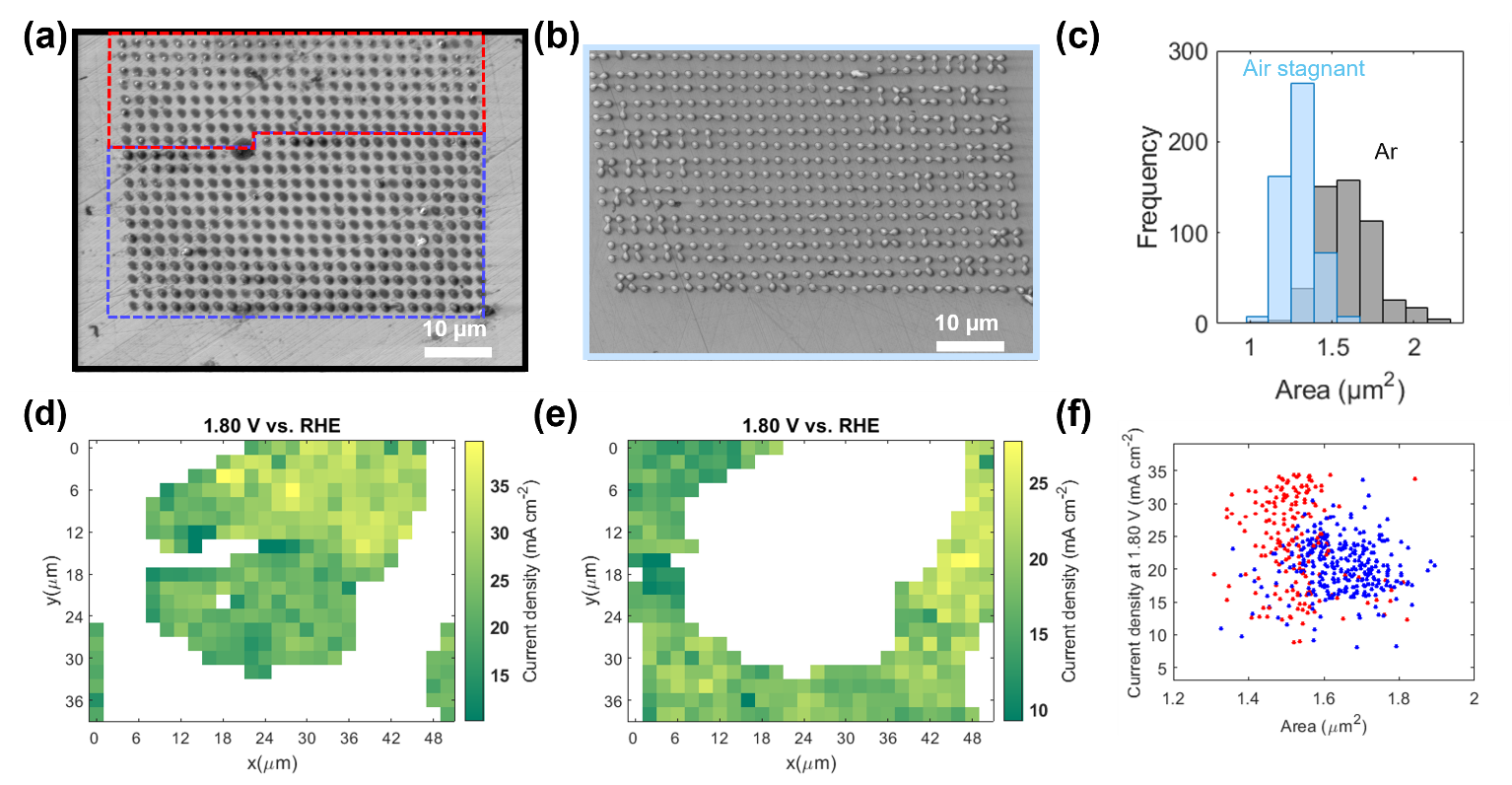


**Figure S5.** (a) and (b) SEM images of the SECCM footprint after experiments with Ar and stagnant air atmospheres, respectively. (c) Histograms showing the geometric area of the footprints observed in the SEM images. Average area: Air stagnant: 1.30 ± 0.01 µm²; Ar: 1.60 ± 0.01 µm². MAs that were analyzed in air stagnant (n = 520) and Ar (n = 514). (d) and (e) Current density maps at 1.8 V vs. RHE for the spots measured in Ni_3_B and Ni_2_B regions, respectively. The activity maps were plotted separately for each Ni-B phase to better visualize the changes in the current density across the scan. (f) Scatter plots showing the variation of the current density with the footprint area grouped by the regions indicated in (a). The red polygons enclose the MAs before the significant increase in the footprint is observed in SEM. The LSVs were recorded in a 0.1 M KOH electrolyte with a 1.0 V s^-1^ scan rate. A 2 µm hopping distance and an environmental chamber with continuously flowing Ar (30 mL min^-1^) were used. Data are presented as mean ± 95 % confidence interval.

**S2.3 Measurement under flowing air**


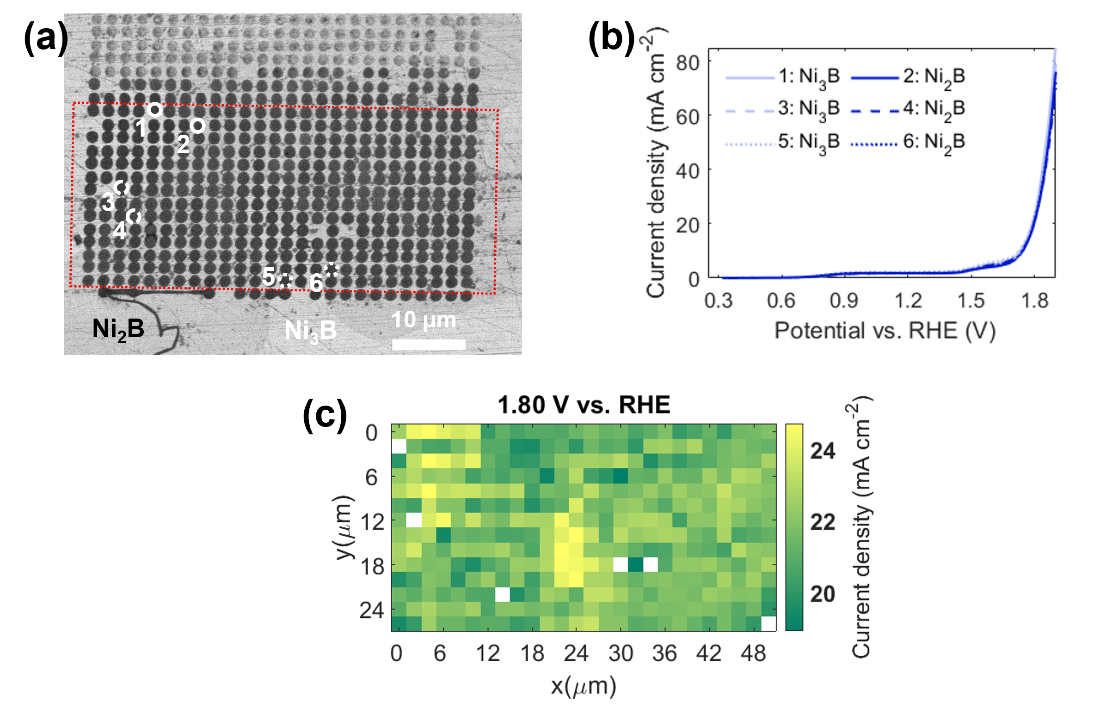


**Figure S6.** (a) SEM image of the Ni_70_B_30_ surface after the SECCM scan using air flow and a ~ 449 nm diameter nanopipette (Figure S2e). (b) Polarization curves of three marked points on the Ni_2_B and Ni_3_B, the current densities were calculated by normalization of the recorded currents to the footprint area calculated from the SEM image. (c) The electrochemical activity map at 1.8 V vs. RHE and (d) corresponding histograms showing the current densities distribution recorded at 1.8 V vs. RHE grouped by phases (light violet -Ni_3_B, dark violet – Ni_2_B). The LSVs were recorded in a 0.1 M KOH electrolyte with a 1.0 V s^-1^ scan rate. A 2 µm hopping distance and an environmental chamber with compressed air continuously flowing at 30 mL min^-1^ were used.

**S2.4 Measurement under O_2_ flow**


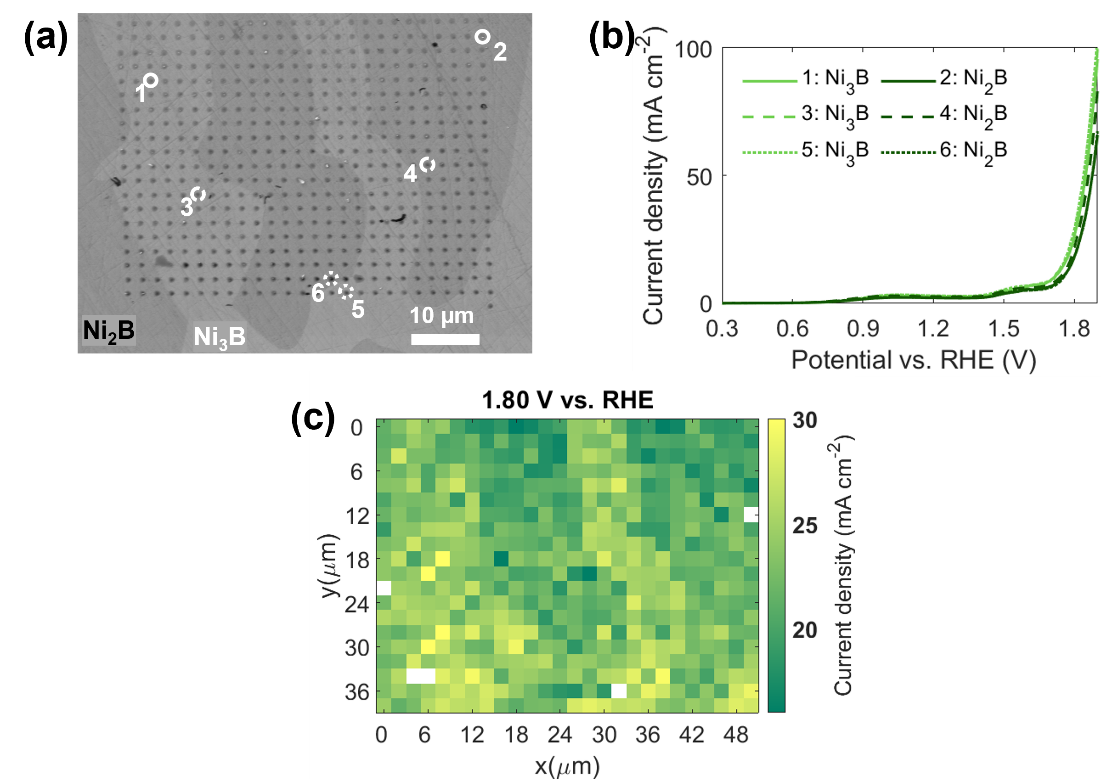


**Figure S7.** (a) SEM image of the Ni_70_B_30_ surface after the SECCM scan using O_2_ and a ~ 394 nm diameter nanopipette (Figure S2c). (b) Polarization curves of three marked points on the Ni_2_B and Ni_3_B, the current densities were calculated by normalization of the recorded currents to the footprint area calculated from the SEM image. (c) The electrochemical activity map at 1.8 V vs. RHE. The LSVs were recorded in a 0.1 M KOH electrolyte with a 1.0 V s^-1^ scan rate. A 2 µm hopping distance and an environmental chamber with compressed air continuously flowing at 30 mL min^-1^ were used.

**S2.5 Measurement under CO_2_-containing Ar atmosphere**


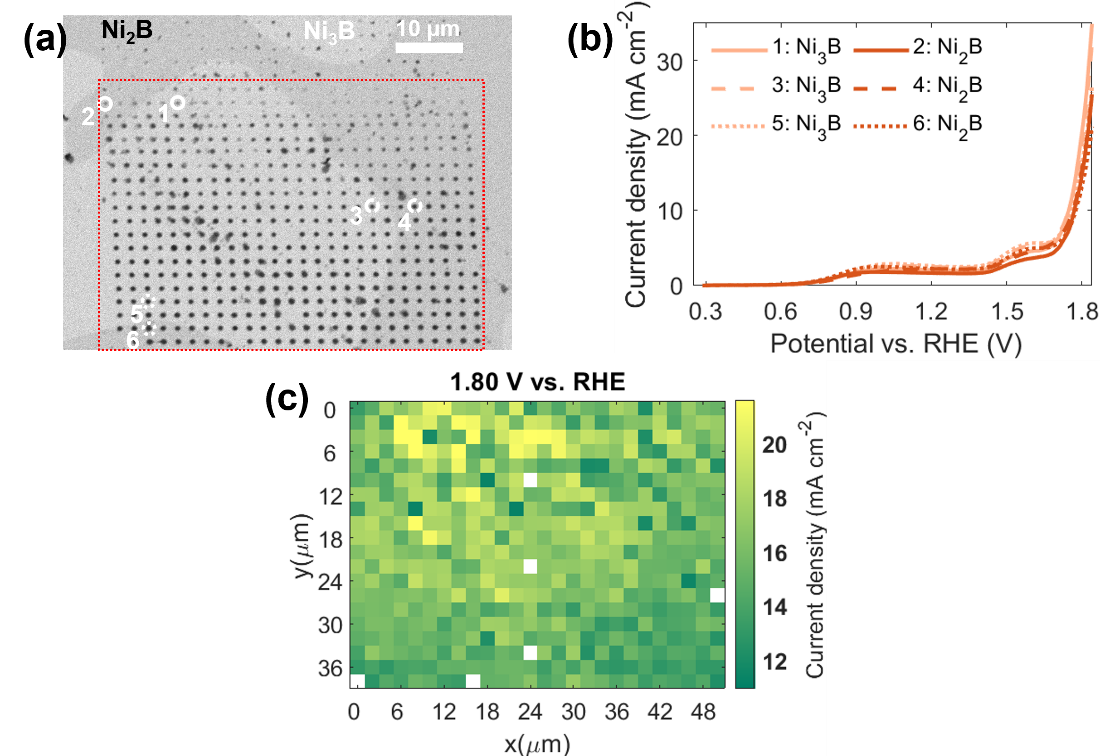


**Figure S8.** (a) SEM image of Ni_70_B_30_ sample after the SECCM scan using the flow of 2 % CO_2_ in Ar and a ~ 396 nm diameter nanopipette (Figure S2d). (b) Polarization curves of three marked points on Ni_2_B and Ni_3_B, the current densities were calculated by normalization of the recorded currents to the footprint area calculated from the SEM image. (c) The electrochemical activity map at 1.8 V vs. RHE and (d) corresponding histograms showing the current densities distribution recorded at 1.8 V vs. RHE grouped by phases (light orange -Ni_3_B, dark orange – Ni_2_B). The LSVs were recorded in a 0.1 M KOH electrolyte with a 1.0 V s^-1^ scan rate. A 2 µm hopping distance and an environmental chamber with 2% CO_2_ diluted in Ar (flow rate of 30 mL min^-1^) were used.

**S2.6 OER activity in different gas atmospheres Ni_2_B and Ni_3_B**


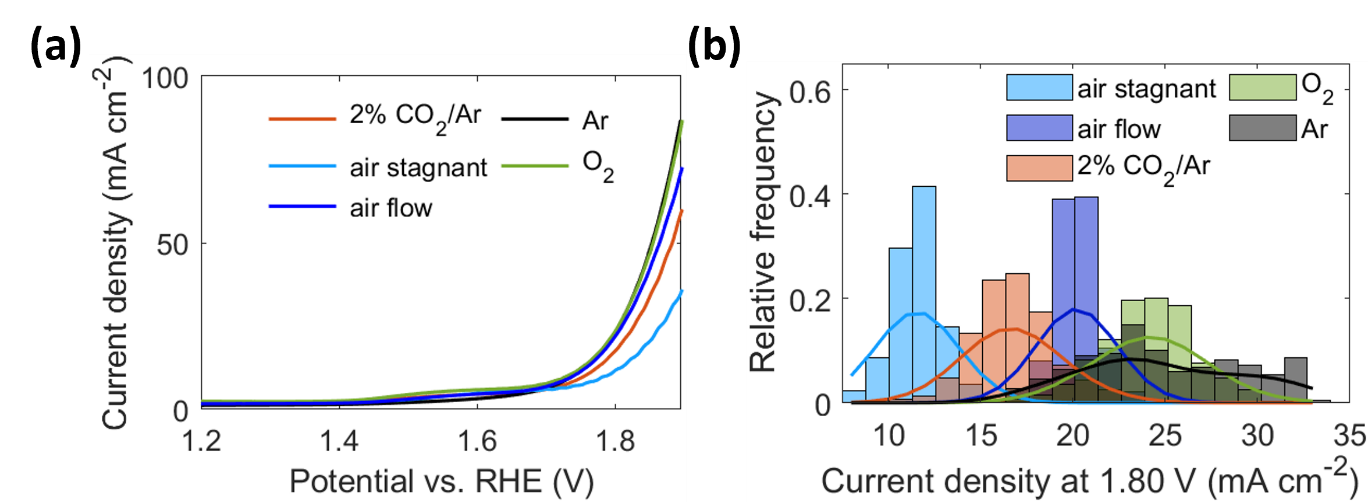


**Figure S9.** (a) Averaged polarization curves recorded over all spots assigned to Ni_3_B under different atmospheres (stagnant air, flowing air, 2% CO_2_/Ar, O_2_ and Ar). (b) Histograms with the current density distribution recorded at 1.8 V vs. RHE on Ni_3_B grouped by the gas atmosphere used during the experiment. MAs analyzed in air stagnant (n = 209), air flow (n = 80), 2% CO_2_/Ar (n = 322), O_2_ (n = 234), and Ar (n = 266).


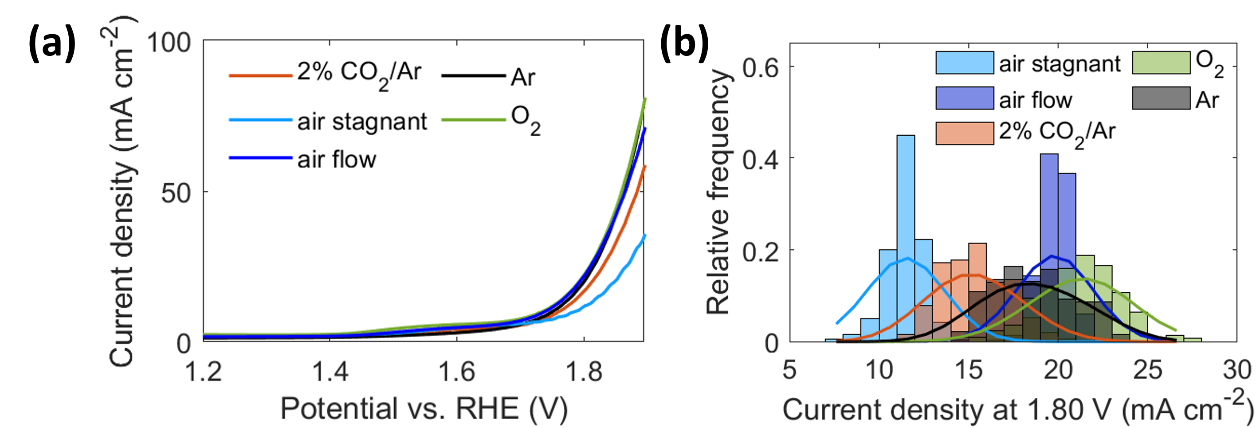


**Figure S10.** (a) Averaged polarization curves recorded over all spots assigned to Ni_2_B at 1V s^-1^ in 0.1 M KOH electrolyte under different atmospheres (stagnant air, flowing air, 2% CO_2_ in Ar, O_2_ and Ar). (b) and (c) Histograms with the current density distribution recorded at 1.8 and 1.9 V vs. RHE on Ni_2_B phase grouped by the atmosphere used during the experiment. MAs analyzed in air stagnant (n = 313), air flow (n = 272), 2% CO_2_/Ar (n = 192), O_2_ (n = 282), and Ar (n = 243).


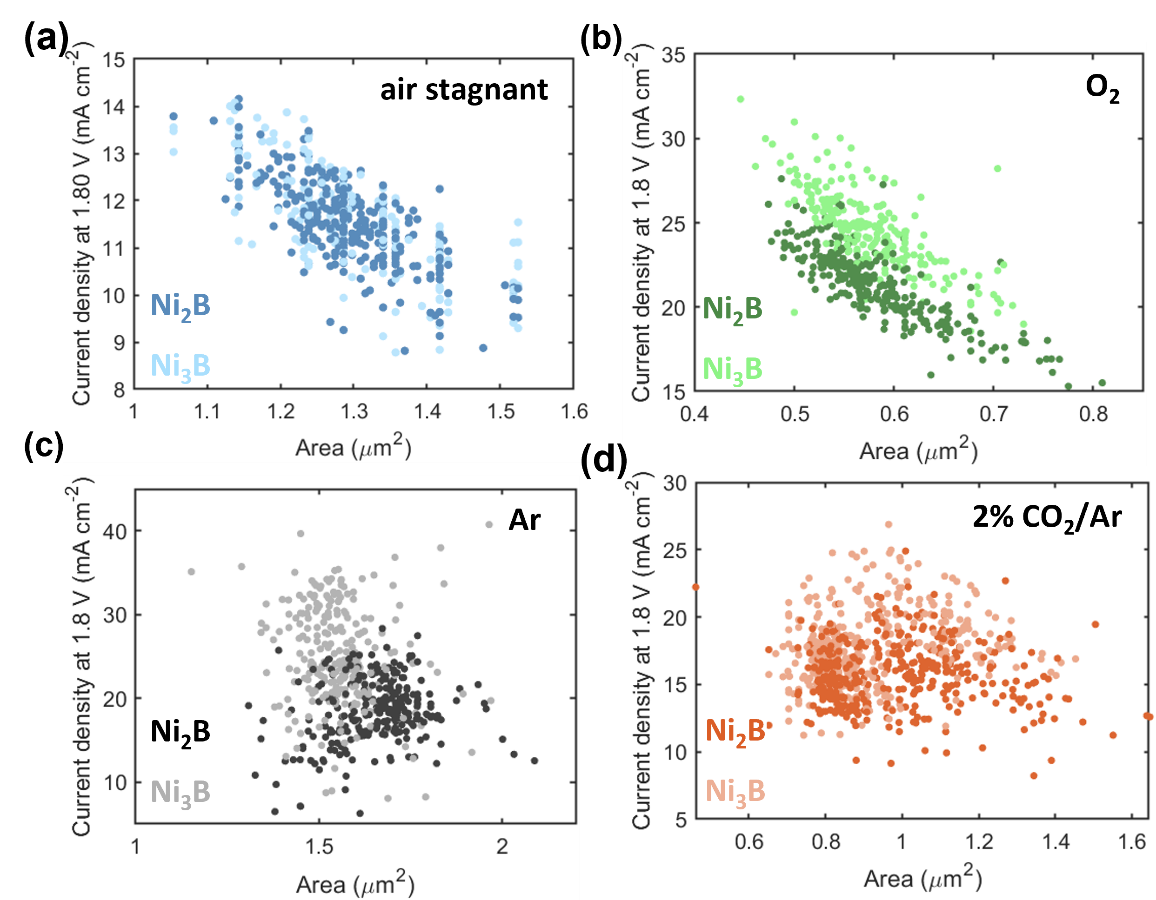


**Figure S11.** Scatter plots of the current densities recorded at 1.8 V vs. RHE as a function of the footprint area of the MAs (based on the processing of the SEM image using ImageJ) grouped by phases (light colored Ni_3_B and dark colored Ni_2_B) during experiments performed under (a) stagnant air, (b) O_2_, (c) Ar and (d) 2% CO_2_ in Ar.

**S2.7 OER activity of Ni_2_B and Ni_3_B in Ar and air on a freshly polished Ni_70_B_30_ ingot**


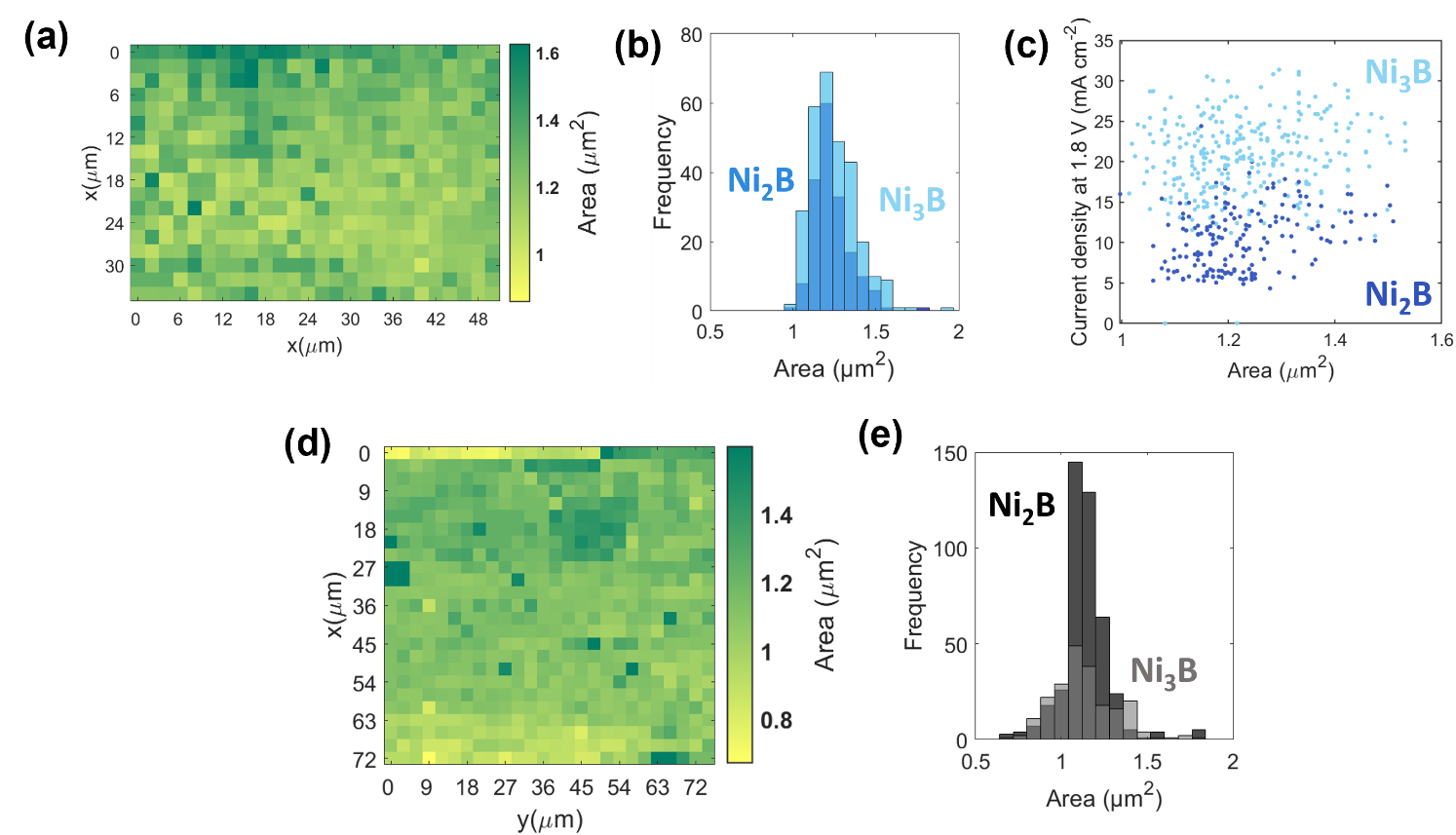


**Figure S12.** Footprint areas of the MAs during the SECCM scan on freshly polished Ni_70_B_30_ surface that were used for current normalization under (a) stagnant air atmosphere and (d) Ar flow. Footprint area distribution grouped by Ni-B phases according to the SEM image for the experiment under (b) stagnant air (Ni_2_B = 1.23 ± 0.02 µm^2^ (n = 175) and Ni_3_B = 1.25 ± 0.02 µm^2^ (n = 293)) and (e) Ar flow (Ni_2_B = 1.14 ± 0.01 µm^2^ (n = 435) and Ni_3_B = 1.13 ± 0.02 µm^2^ (n = 215)). (c) Current density vs. footprint area correlation derived for the Ni_2_B (dark blue) and Ni_3_B (light blue). Data are presented as mean ± 95 % confidence interval.

**References**

[1] *WinXPow (Version 2.25)*; STOE and Cie GmbH: Darmstadt, Germany, 2003.

[2] L. Akselrud, Yu. Grin WinCSD: Software Package for Crystallographic Calculations (Version 4), J. Appl. Crystallogr. **2014**, 47, 803.

[3] *Olympus Stream Enterprise Desktop Version 2.3.3*; Olympus Soft Imaging Solutions GmbH, Münster, Germany.

[4] J. L. Pouchou, F. Pichoir A new model for quantitative X-ray microanalysis. Part I: application to the analysis of homogeneous samples, Rech. Aerospatiale **1984**, 3, 13.

[5] O. J. Wahab, M. Kang, P. R. Unwin *Scanning electrochemical cell microscopy: A natural technique for single entity electrochemistry*, *Curr. Opin. Electrochem.* **2020**, *22*, 120.

[6] D. Martín-Yerga, P. R. Unwin, D. Valavanis, X. Xu *Correlative co-located electrochemical multi-microscopy*, *Curr. Opin. Electrochem.* **2023**, *42*, 101405.
